# Microstructure and Properties of Polypropylene/Carbon Nanotube Nanocomposites

**DOI:** 10.3390/ma3042884

**Published:** 2010-04-21

**Authors:** Dimitrios Bikiaris

**Affiliations:** Laboratory of Polymer Chemistry and Technology, Department of Chemistry, Aristotle University of Thessaloniki, 541 24, Thessaloniki, Macedonia, Greece; E-Mail: dbic@chem.auth.gr; Tel.: 30 2310 997812; Fax: 30 2310 997667

**Keywords:** nanocomposites, polypropylene, carbon nanotubes, physical properties

## Abstract

In the last few years, great attention has been paid to the preparation of polypropylene (PP) nanocomposites using carbon nanotubes (CNTs) due to the tremendous enhancement of the mechanical, thermal, electrical, optical and structural properties of the pristine material. This is due to the unique combination of structural, mechanical, electrical, and thermal transport properties of CNTs. However, it is well-known that the properties of polymer-based nanocomposites strongly depend on the dispersion of nanofillers and almost all the discussed properties of PP/CNTs nanocomposites are strongly related to their microstructure. PP/CNTs nanocomposites were, mainly, prepared by melt mixing and *in situ* polymerization. Young’s modulus, tensile strength and storage modulus of the PP/CNTs nanocomposites can be increased with increasing CNTs content due to the reinforcement effect of CNTs inside the polymer matrix. However, above a certain CNTs content the mechanical properties are reduced due to the CNTs agglomeration. The microstructure of nanocomposites has been studied mainly by SEM and TEM techniques. Furthermore, it was found that CNTs can act as nucleating agents promoting the crystallization rates of PP and the addition of CNTs enhances all other physical properties of PP. The aim of this paper is to provide a comprehensive review of the existing literature related to PP/CNTs nanocomposite preparation methods and properties studies.

## 1. Introduction

Isotactic polypropylene (iPP) is one of the most important commodity thermoplastics, accounting for about 20% of the total world polyolefin production [1]. Its high isotacticity, enhanced mechanical properties, narrowmolecular weight distribution and clarity lead to a balance of physical and mechanical properties, and its environmental friendliness (recyclability) and low cost give it an extra advantage. Due to its low cost, low density, high thermal stability and resistance to corrosion, iPP is widely used in many applications, such as fibers, films for food packaging, production of bottles and tubes, *etc.* However, despite all these advantages, there is also a drawback in its applications. Although its resistance to crack initiation is very high, in crack propagation the resistance of the iPP matrix is very low; therefore, when a crack or mechanical failure exists, the matrix can break very easily. This is especially an issue at low temperatures. Therefore, a great deal of effort has been made to modify its mechanical properties such as blending iPP with inorganic fillers in the form of nanoparticles. Furthermore, these inorganic nanofillers, such as talc, silicon dioxide, carbon black, clay, and carbon nanotubes, act as nucleating agents for PP crystallization as well as they enhance its physical properties.

With the rapid development of nanotechnologies and nanomaterials since 1990s, studies on polymer-based nanocomposites have been extensively carried out in order to find promising alternatives to traditional composites, though these mainly focused on general mechanical and multifunctional properties and filler dispersion. In the case of PP nanocomposites, the most used filler is carbon nanotubes (CNTs). This is due to the unique combination of structural, mechanical, electrical, and thermal transport properties of carbon nanotubes (CNTs) [2], obtaining advanced multifunctional composite materials [3,4].

**Figure 1:**
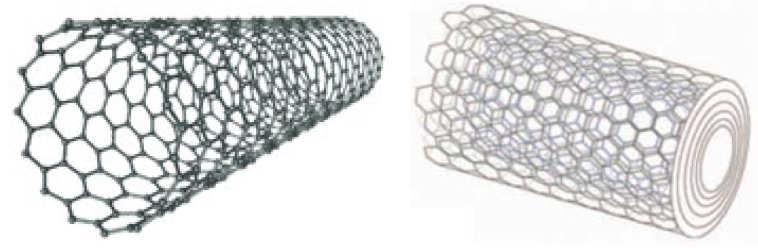
Illustration of SWCNTs (left) and MWCNTs (right).

Carbon nanotubes are categorized as single-walled nanotubes (SWCNTs), multi-walled nanotubes (MWCNTs) and double-walled nanotubes (DWCNTs), which are of minor importance (Figure 1). Most SWCNTs have a diameter of close to 1 nm, with a tube length that can be many thousands of times longer. The structure of SWCNTs can be conceptualized by wrapping a one-atom-thick layer of graphite, called graphene, into a seamless cylinder. SWCNTs are a very important variety of CNTs because they exhibit important electric properties that are not shared by the MWCNTs variants. The MWCNTs consist of multiple layers of graphite rolled in on themselves to form a tube shape. MWCNTs, however, have a lower tensile strength and modulus than SWCNTs. Furthermore, SWCNTs have a shorter diameter (from 0.4 up to 5.6 nm) compared with MWCNTs, which is in the range of 10-100 nm. MWCNTs are also more rigid, because their section is much larger compared to that of SWCNTs.

MWCNTs and SWCNTs have been considered as unique reinforcements for different polymers due to their exceptional electrical, thermal, chemical and mechanical properties. The tensile strength, tensile modulus and Poisson ratio for SWCNTs have been reported to be in the range of 37–100 GPa, 640 GPa to 1–2 TPa [5] and 0.14–0.28 [6], respectively. For comparison, the modulus of graphite fibers and steel are in the range 300–800 and 200–400 GPa, respectively, and the tensile strengths of these materials are about 5 and 10–20 GPa, respectively, whereas the density of SWCNTs is one-sixth that of steel. Furthermore, this is coupled with approximately 500-times more surface area per gram (based on equivalent volume fraction of typical carbon fiber) and an aspect ratio of around 100-1000.

Investigations of PP nanocomposites fabricated with CNTs have been reported by many researchers [7,8,9,10]. However, it is well-known that the properties of polymer-based nanocomposites are strongly dependent on the dispersion of nanofillers.

The microstructure of the PP/CNTs nanocomposites is discussed in the present paper, while emphasis is given to how this microstructure affects the properties of the final material.

## 2. Synthesis of CNTs

CNTs are sheets of graphite rolled into tubes and have excellent properties due to their symmetric structure. For the synthesis of CNTs there are three main synthetic methods used: Arc-discharge, laser ablation and catalytic methods such as chemical vapor deposition [11]. Synthesis procedures yielding high purity nanotubes of defined structure are still restricted to carbon arc discharge and pulsed laser ablation, allowing gram per day rather than kilograms per day production quantities. Higher quantities can be manufactured by chemical vapor deposition processes, having reasonable potential for extended industrial production but yielding materials with higher defect density.

### 2.1. Arc-discharge

Arc-discharge is the easiest and most common method of producing CNTs. This technique involves the growth of CNTs on carbon (graphite) electrodes during the direct current arc-discharge evaporation of carbon in the presence of an inert gas such as helium or argon. The method is similar to that used for the fullerene synthesis. Plasma is created between anode and cathode. The temperature of this plasma typically reaches temperatures of approximately 3700-4000 °C. At this temperature, the carbon on the anode is vaporized and deposits onto the cathode. It should be noted that the diameter of the anode is usually smaller than the diameter of the cathode and both electrodes are water-cooled. The carbon needles, ranging from 4 to 30 nm in diameter and up to 1 mm in length, were grown on the negative end of the carbon electrode used for the direct current arc-discharge evaporation of carbon in an argon-filled vessel.

### 2.2. Laser ablation

According to this method, in a 1200 °C furnace a pulsed or a continuous laser is used to vaporize a target consisting of a mixture of graphite and metal catalysts, such as cobalt or nickel, in the presence of 500 Torr (67 kPa) helium or argon gas. As the target is vaporized, a cloud of C_3_, C_2_, C and catalyst vapors is formed rapidly. As the cloud cools, the carbon species with a small molecular weight then combine to form larger molecules. The vaporized catalysts condense slowly and adhere to carbon clusters to prevent their closing into cage structures. The nanotube grows until too many catalyst atoms aggregate on the end of the nanotube. The large particles either detach or become over-coated with sufficient carbon to poison the catalysis. This allows the tube to terminate with a fullerene-like tip or with a catalyst particle. With this method can achieve high yields (>70%) of SWCNTs.

### 2.3. Chemical Vapor Deposition (CVD)

This method is capable of controlling growth direction on a substrate and synthesizing a large quantity of nanotubes. The CVD technique involves the decomposition of a hydrocarbon in the presence of a catalyst. In this process a mixture of hydrocarbon gas, acetylene, methane or ethylene and nitrogen is introduced into the reaction chamber. During the reaction, nanotubes are formed on the substrate by the decomposition of the hydrocarbon at temperatures 700–900 °C and atmospheric pressure. The most common catalysts used for CVD are iron, nickel or cobalt. Carbon has a low solubility in these metals at high temperatures and thus the carbon will precipitate to form nanotubes.

## 3. PP/CNTs Nanocomposites

There are three main processing methods used for the preparation of PP nanocomposites with nanotubes:

**a)**  The solution method; where both the polymer and nanoparticles are dispersed in an organic solvent and nanocomposites are produced in the form of film after solvent evaporation.
**b)**  *In situ* polymerization; where both nanoparticles and monomers are mixed from the beginning of polymerization.
**c)**  Melt mixing; where nanoparticles are added in the molten polymer at temperatures of 30-60 °C above the melting point of the polymer.

Since the solubility of polypropylene is too low in most organic solvents, solution casting is not applied for the preparation of nanocomposites and melt mixing as well as *in situ* polymerization are the most used methods. Melt mixing can be done in a variation of different types of machines and is a very easy and most common method. However, the nanocomposites prepared by melt mixing or *in situ* polymerization have completely different characteristics as well as properties, which will be discussed in following.

### 3.1. Microstructure of PP/CNTs nanocomposites

Due to the CNTs intrinsic poor solubility, they are often self-assembled into bundles, which limits their applications. Bulk agglomerates of nanotubes up to several tens of microns in diameter act as macro-particle like centres of friction, unable to be compensated by regions exhibiting proper dispersion of nanotubes within the composite where physical properties are strongly enhanced. CNTs have high aspect ratio and surface reactive groups which prompt the interactions between nanotubes and thus very difficult can be separated and intercalated into polymer matrix. Good control over various mixing parameters during melt mixing is essential to obtain homogeneous composite materials. The microstructure and CNTs dispersion into PP matrix is very crucial in order a high performance material to be achieved. Thus, there are many research groups that focus on study of the PP/MWCNTs microstructure using different analytical techniques such as Scanning Electron Microscopy (SEM), Transmission Electron Microscopy (TEM), Atomic Force Microscopy (AFM), Polarizing Optical Microscopy (POM), micro-Raman spectroscopy, *etc.*

**Figure 2:**
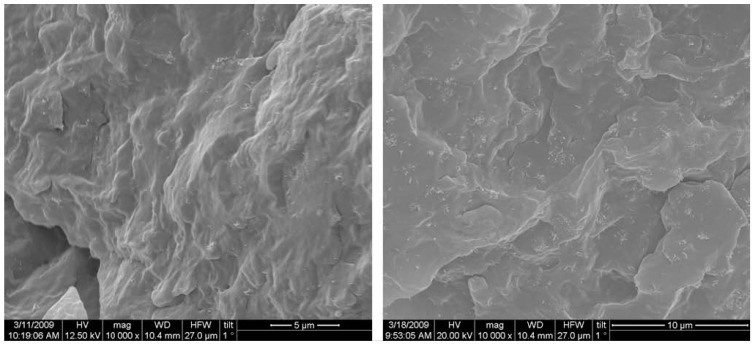
SEM micrographs for the nanocomposites containing 1 wt % (left) and 3 wt % (right) MWCNTs. Reproduced with permission from ref. [13].

The microstructure of iPP/MWCNTs nanocomposite fractured surfaces studied by SEM analysis reveals different filler dispersion efficiency achieved in various composite systems [12,13]. From the micrographs in Figure 2, it can be seen that composites with different MWCNTs contents exhibited different MWCNTs dispersion state. At low MWCNT content, most of the MWCNTs dispersed individually in the iPP matrix. At relatively high MWCNTs contents, MWCNTs aggregates appeared in the polymer matrix, and the size of the aggregates increased with increasing MWCNTs content. [10,14]. Solid-state shear pulverization in conjunction with melt mixing was found that can produce well-dispersed polymer/MWCNTs nanocomposites [15,16].

**Figure 3:**
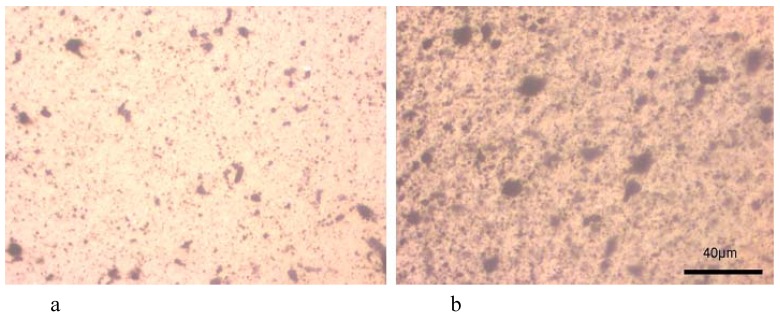
Optical micrographs of PP/MWCNTs nanocomposites containing different MWCNTs content. a) 0.5 wt % and b) 2.5 wt %.

The agglomeration of nanotubes in clusters or clumps in the nanocomposite can be seen more clearly in an optical microscopy micrograph (Figure 3) [17,18]. As can be seen CNTs aggregates are observed as dark spots with a wide particle size distribution. Particles with sizes 0.5 up to 20 μm are visible. The particle sizes are mainly depended on the CNTs concentration. At low MWCNTs content they formed agglomerates are in lower magnitude and a better dispersion can be seen. The mean particle sizes are in the range of 0.2 up to 10 μm, while as MWCNTs content increases nanotubes forms larger agglomerates. This is due to the tendency of nanotubes to interact each other due to surface forces. CNTs during their formation have surface carboxyl groups and maybe some hydroxyl groups. These interact through hydrogen bonding or due to weak Van der Wall’s forces creating clusters and agglomerates. These are very difficult to break down during nanocomposites fabrication, since the involved forces during melt extrusion are not enough to break these agglomerates.

**Figure 4:**
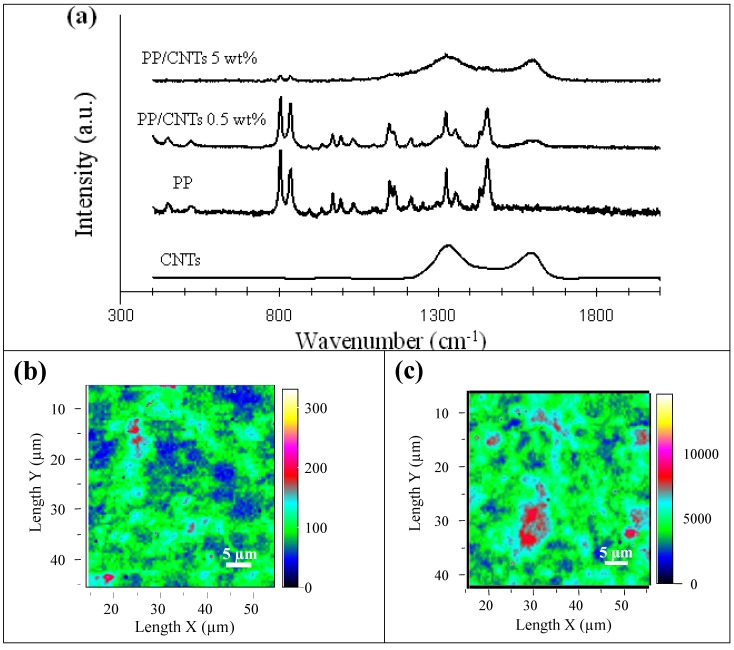
(a) Raman spectra of CNTs, neat iPP, and composites at 0.5 and 5 wt % CNTs content. (b) and (c) Micro-Raman maps from samples containing 0.5 wt % and 5 wt % CNTs, respectively. The color bar indicates the intensity of the peak at 1594 cm^-1^.

Visualization of the MWCNTs aggregates was also attempted by means of confocal micro-Raman spectroscopy [18]. Their identification is based on the absorption of PP and MWCNTs at completely different wavenumbers. As can be seen in Figure 4a, the MWCNTs spectrum exhibits a distinct pair of peaks at 1573 (major) and 1602 (shoulder) cm^-1^, which does not conflict with peaks from iPP. In the nanocomposites both the characteristic peaks of MWCNTs and PP are recorded. When plotted with respect to the peak intensity at 1573 cm^-1^, 2D micro-Raman scans acquired from iPP/MWCNTs samples can provide information on the size of the nanotube aggregates. Red colors indicate very high concentration of MWCNTs while the blue color very low. As can be seen in Figure 4b there are a lot of areas that red color has high intensity indicating that MWCNTs formed aggregates with different sizes. Aggregates with sizes in the area of 0.5 μm are visible as well as particle sizes with diameters till 5 μm. These observations were consistent for all the samples examined and in agreement with POM that was discussed previously and those from TEM images. Thus, the supporting information provided from confocal micro-Raman spectroscopy, which enabled scanning of larger areas and along various planes within the sample, lead us to believe that the findings of the TEM examination can be considered representative of the samples studied.

**Figure 5:**
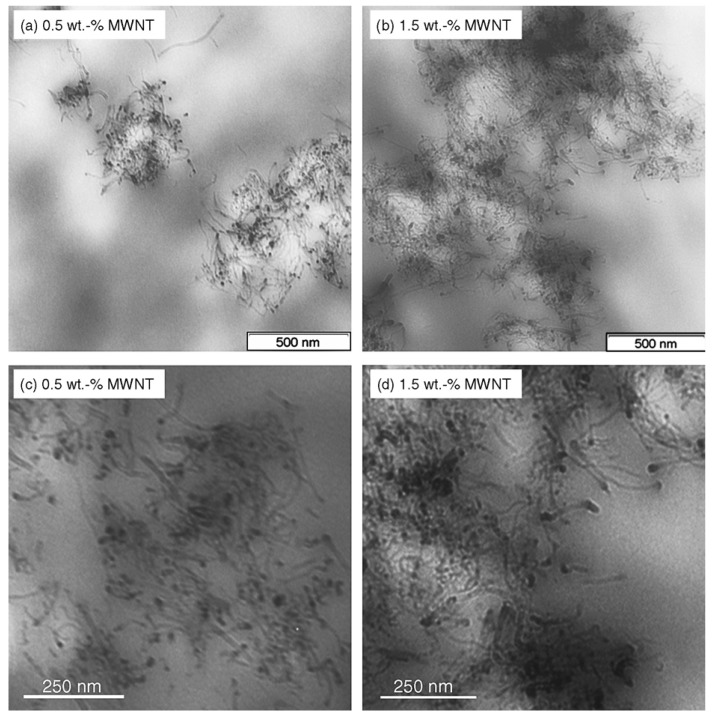
Dispersion of the MWCNTs: TEM image of the nanocomposite with a) 0.5 and b) 1.5 wt.% of MWCNTs at low magnification and c) TEM image of the nanocomposite with 0.5 and (d) 1.5 wt.% of MWCNTs at high magnification. Reproduced with permission from ref. [19].

From the above presented images, it could be wrongly concluded that CNTs are dispersed into PP matrix always in the form of aggregates, while individual CNTs do not exist. However, this is not true, since SEM or POM techniques have low resolution, and thus TEM is more appropriate to study efficiently the microstructure and the CNTs dispersion in a polymer matrix. TEM-images of the PP/MWCNTs nanocomposites containing 0.5 and 1.5 wt % MWCNTs (Figure 5) reveal indeed that these agglomerates observed by SEM and POM techniques are formed from large numbers of nanotubes [19]. However, as can be seen in all agglomerates, the powdery nanotube aggregates (prior to mixing) were infiltrated by the polymer matrix during the melt-mixing procedure and, thus, the diffusion of polymer chains have widened the distance between the individual nanotubes. Furthermore, areas with higher CNTs concentration are seen next to nearly CNTs free regions in the TEM figures at low magnification indicating partially incomplete distribution, though qualitatively there is a relative good state of dispersion at low MWCNTs contents (Figure 5a and c). The dispersion of MWCNTs as individual nanotubes into polymer matrix is very difficult, since the involved interactions between PP macromolecules and nanotubes should be stronger than the interactions between nanotubes. Some individual CNTs can be seen only at low CNTs concentration (0.5 wt %) since the tendency for agglomeration is increased at higher MWCNTs contents (>1.5 wt %).

**Figure 6:**
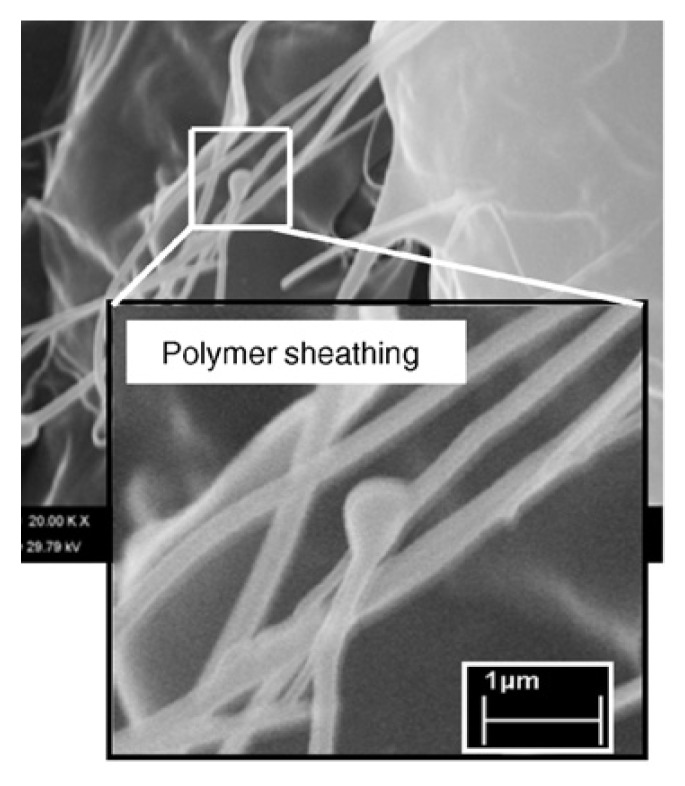
SEM micrograph of ‘‘polymer sheathing” phenomena of the fractures surface of PP/MWCNTs nanocomposite with 1 wt % of MWCNTs. Reproduced with permission from ref. [19].

From the above TEM images it can be seen that during mixing with PP, carbon nanotubes have a small tendency to be separated and dispersed homogenously into PP matrix at low CNTs concentration. This is due to the small interfacial adhesion between the carbon nanotubes and PP matrix as was confirmed by SEM microscopy. From SEM micrograph (Figure 6) corresponding to the fractured surfaces of nanocomposite with 1 wt % of MWNTs, it is observed that the nanotubes appear to have higher diameters at their broken/debonded ends as compared to other parts, indicating that the polymer chains prior to failure are stretched to their maximum followed by recoiling and balling-up around the tip of the broken end of the nanotubes. The outer polymer sheath, in such a case, gets contracted and balled up during fracture process [19].

**Figure 7:**
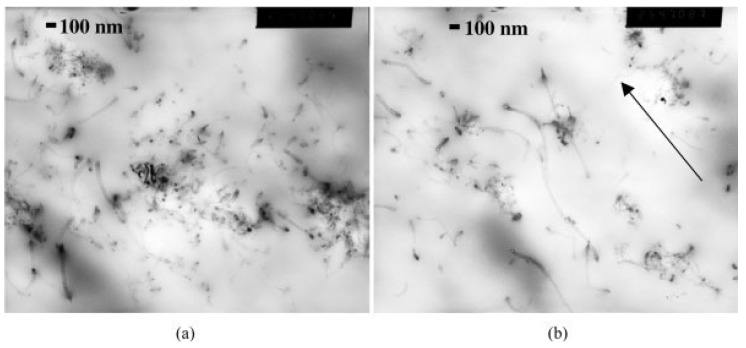
TEM Images of 3 wt % MWNTs in PP a) un-oriented; b) oriented, 12:1 draw ratio. Arrow indicates the direction of orientation. Reproduced with permission from ref. [8].

The dispersion of CNTs into PP matrix may be also affected from the applied draw during the melt procedure [8]. Figure 7 shows TEM images of a 3 wt % MWCNTs in PP both un-oriented (no draw) and oriented at 12:1 draw ratio. These images depict the cross section of the fiber parallel to the drawing direction. Comparing the two images reveals how melt drawing the nanocomposite orients the nanotubes. In the un-oriented image (Figure 7a), whole nanotubes lying at various oblique angles are observed; whereas, the image of the drawn sample (Figure 7b) shows nanotubes that run parallel to the fiber axis (as shown by the arrow), therefore indicating flow induced orientation along the fiber axis.

Except for the MWCNTs content, the kind of the PP used also plays an important role for MWCNTs dispersion into PP matrix. In a recent study, both individual nanotubes and nanotube aggregates were present in iPP and sPP matrices [12]. However, a much higher degree of CNT agglomerate exfoliation has been attained in iPP/MWCNTs nanocomposites. sPP-based nanocomposites exhibit strong filler aggregation, while well-separated individual MWCNTs prevail of use iPP/MWCNTs materials. For the sPP/0.4 wt.% MWCNTs sample, predominant formation of relatively compact nanotube clusters integrating 6–8 individual nanotubes takes place, but at filler contents higher than 1.5 wt.% strongly agglomerated CNT structures with micrometer dimensions are formed in sPP matrix. Due to considerable nanotube aggregation, the sPP/MWCNTs materials have substantially lower interfacial area as compared to the respective iPP/MWCNTs nanocomposites.

All the above mentioned studies concerning the microstructure of PP/CNTs nanocomposites revealed that CNTs can form aggregates into PP matrix. These are very difficult to destroy during melt mixing and CNTs dispersed intercalated into PP matrix. It seems that the applied shear forces are not strong enough to destroy the nanotube agglomerates. This microstructure directly affects most of the physical properties of PP/CNTs composites and mainly the mechanical properties. At low CNTs concentration an enhancement is observed, while higher CNTs concentrations cause deterioration. This will be discussed in the following.

### 3.2. Mechanical properties

The combination of enormous modulus (~1 TPa) and the high aspect ratio (100–1000) of CNTs makes them potentially effective as reinforcement for polymers. Thus, the incorporation into PP matrix will enhance its mechanical properties [20,21]. Due to the nanoscale dimensions of the carbon nanotubes the interfacial regions surrounding the tubes are also in nanometer dimensions and the applied load can be easily transferred from matrix to CNTs. There are three synergizing aspects of interaction in carbon-nanotube-based nanocomposites, *i.e.*, polymer–nanotube interaction, nanotube–nanotube and intra-polymer interactions in polymer-carbon nanotube interactions. It was observed that continuous nanotubes most effectively enhance the buckling resistance of the composites [22]. Despite an increasing thrust in the area of carbon-nanotube-filled polymer nanocomposites, the mechanism and the magnitude of load transfer between polymer matrices and the nanotubes are unclear and research in this area is still in its infancy.

**Figure 8:**
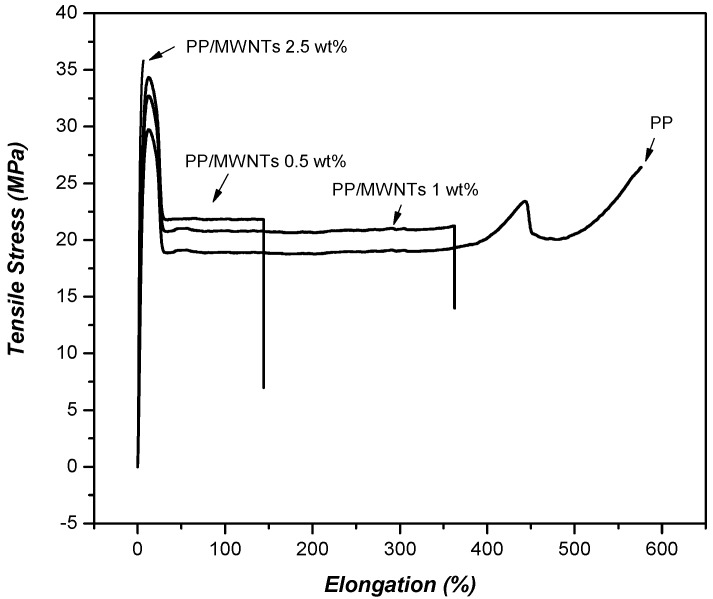
Stress–strain curves of PP/MWCNTs nanocomposites containing different MWNT content.

Typical tensile stress–strain curves of nanocomposite samples with respect to their nanotube content are shown in Figure 8. The stress–strain curves of PP/MWCNTs exhibit distinctly regions of elastic, yielding and plastic deformation accompanied by cold drawing, whereas continued deformation after the yield point resulting in necking along the gauge length. For PP, the typical behavior of a ductile material is observed with a very high elongation at break (620%). The strain hardening region appears at about 400% of elongation and after which tensile strength increases almost linearly with strain until fracture eventually occurs. Also, in case of nanocomposites with lower loading (1 wt % MWCNTs) a ductile behavior is observed with necking, but with lower strain at fracture and without strain hardening. Nanocomposites bearing higher nanotube content (2 wt % MWCNTs) showed a brittle behavior with breaking after the yield point [23,24,25].

**Figure 9:**
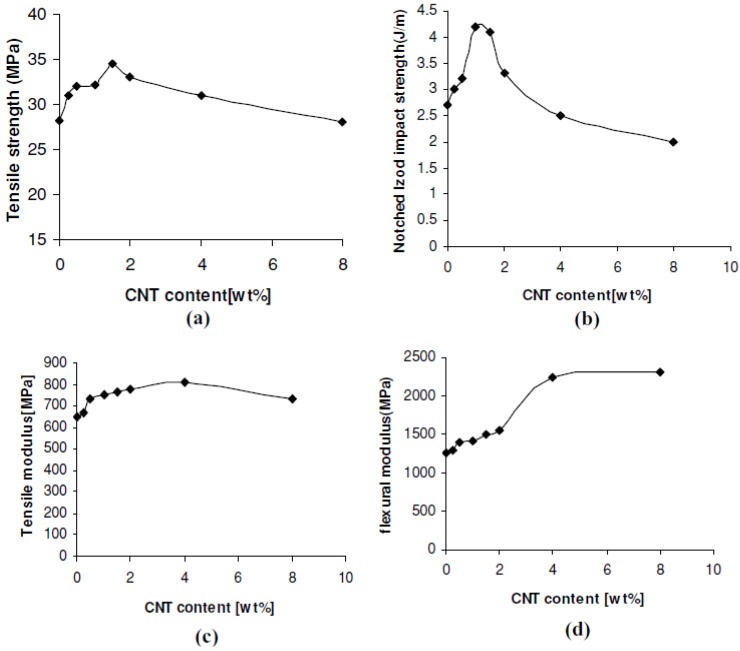
Mechanical properties of PP/CNTs nanocomposites. a) Tensile strength, b) notched Izod impact strength c), tensile modulus and d) flexural modulus. Reproduced with permission from [26].

From stress-strain curves tensile strength and elastic modulus can be calculated. As can be seen in Figure 9, tensile strength and modulus of PP/MWCNTs were increased with increasing MWCNTs content, but after the formation of some aggregates these properties were decreased [26,27]. This is because the size of aggregates increases by increasing the nanotube content. At low CNTs, content nanotubes act as reinforcement agents, but at higher contents the formed aggregates act as mechanical failure concentrators. Thus, PP nanocomposites exhibit their higher mechanical performance at concentration 2-2.5 wt % while with further increase of MWCNTs, the tensile strength of PP/MWCNTs nanocomposites decreases. At low MWCNTs content, partial tensile strain can be transferred to MWCNTs embedded in PP matrix under tensile stress, which leads to the increase of tensile strength. With further addition of MWCNTs, more agglomerates of MWCNTs form in PP matrix and many defects are introduced into the polymer matrix due to the difficulty of homogeneously dispersing MWCNTs by melt mixing. These defects lead to the decrease of tensile strength. However, in all the cases the mechanical properties are higher from that of neat PP indicating the reinforcement effect of MWCNTs on PP matrix. Only elongation at break decreases continuously by increasing CNTs content. This was also verified from stress-strain curves discussed above.

Evidences of stress transfer in polymer–nanotube composites are largely based on enhancement of modulus. Micromechanical analysis of the effective elastic properties based on the Mori–Tanaka approach was also applied, which was later compared with the results from finite-element modelling [28]. Based on these it was concluded that all the axial and transverse elastic properties are dominated by nanotubes and polymer matrix, respectively, while on the other hand the interphase effects greatly impact the transverse properties at low volume fractions. Composites containing 15 wt % fiber filling showed an improvement of the Young’s modulus of about 90%, which means in figures an increase from 410 N/mm^2^ for the pure PP to 780 N/mm^2^ for the nanofiber reinforced composite [29].

**Figure 10:**
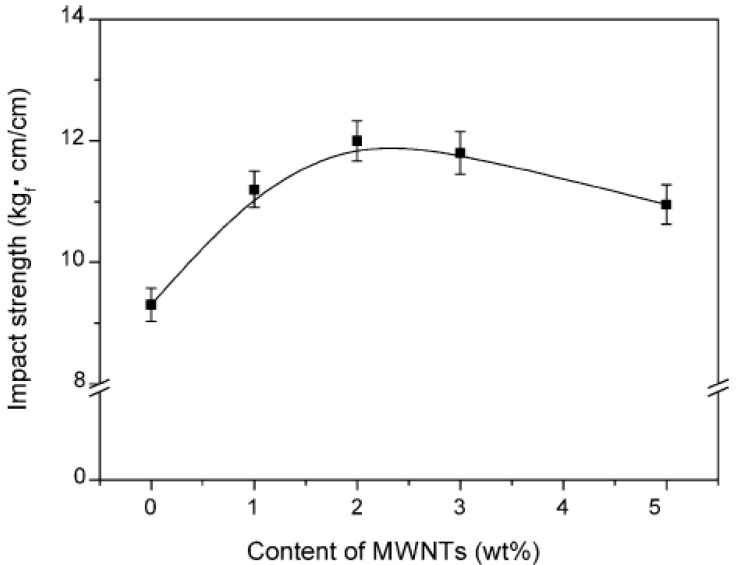
Impact strength of MWCNTs/PP composites as a function of MWNTs content. Reproduced with permission from ref. [30].

Impact strength has also a similar behavior with tensile strength as was reported by Seo *et al.*, [30]. At low MWCNTs concentration, there is a steadily increase reaching the maximum value at 1.5-2 wt % MWCNTs, and gradually decreasing after that load. The extended agglomeration at such high MWCNTs content has a negative effect since the applied load into PP matrix cannot be transferred to MWCNTs. Figure 10 shows the impact strength of PP/MWCNTs composites as a function of MWCNTs content in the PP matrix resins.

The impact strength is improved considerably by the inclusion of just 1 wt % of MWCNTs in the PP/MWCNTs composites and increased with an addition to MWCNTs content, compared with the composites without MWCNTs. In general, the composites with MWCNTs greatly affect the impact damage condition. During induced impact-damage in the composites, the damage zone acts to magnify stress locally because of matrix cracking. However, this failure behavior by impact loading is hindered by the presence of MWCNTs in the composites. Therefore, an increasing fraction of MWCNTs in the composites improves the impact strength, at least up to 10 wt %, which is also largely related to the fracture toughness of the composites. In other words, the stronger the interfacial adhesions, the higher the impact strength. Moreover, the fracture strain of MWCNTs is estimated to be 10–30%, allowing extensive bending and buckling. This high flexibility most probably provides an additional source of higher impact strength to the composites.

**Figure 11:**
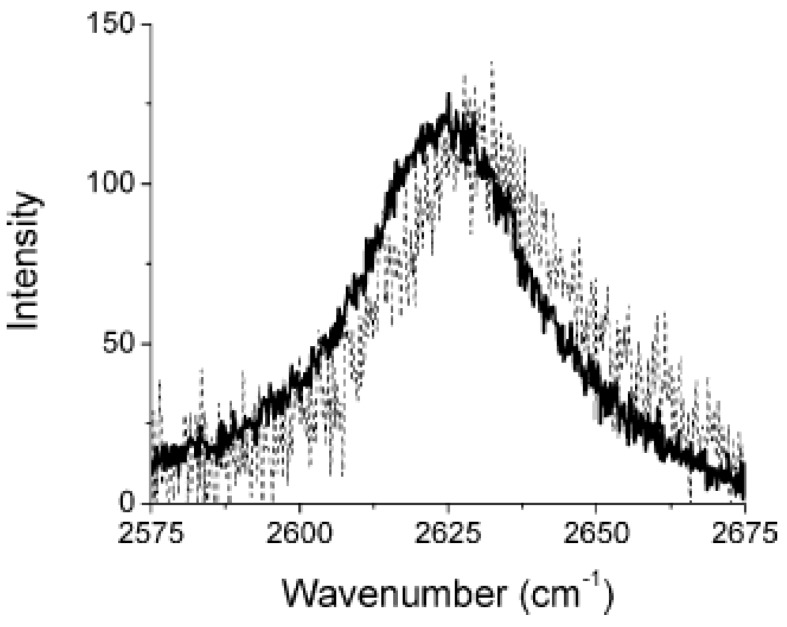
The Raman spectra of D*-mode in PP-1 wt % SWCNTs composite with no strain (dashed line) and with 2% strain (solid line). Reproduced with permission from ref. [27].

Raman spectroscopy has been used to analyze the load transfer from the matrix to the nanotubes [31]. Frequency of SWCNTs D*-band is sensitive to stress and is often used as a stress sensor [27]. Figure 11 shows the spectra of D*-band of the sample containing 1 wt % SWCNTs with no strain and with 2% strain. The mode shifts to lower frequency under tensile stress. The D*-band peak was fit by a Lorentzian curve to estimate the position of its maximum. The peak position of the D*-band is seen to shift to a lower value on application of a tensile strain. The initial slopes are approximately ~200 cm^-1^ per strain. These data demonstrate clear transfer of the load to SWCNTs. The data also show change in the slope at strain above ~0.8–1.0%.

**Figure 12:**
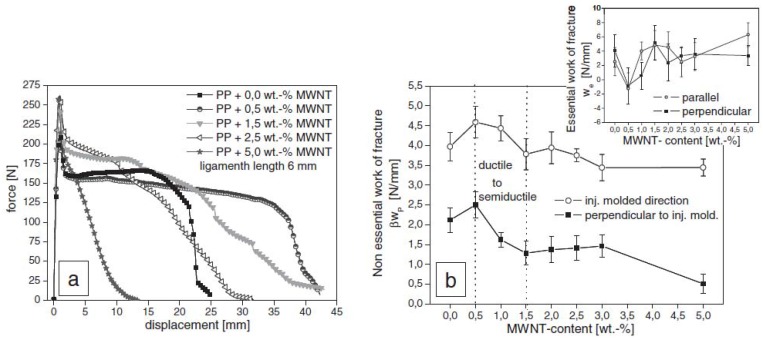
a) Force-displacement diagrams of nanocomposites with different concentrations of MWCNTs for a fixed ligament length (≈6mm). b) Dependence of non-essential work of fracture (*β_Wp_*) on MWCNTs content. Essential work of fracture (*_We_*) *versus* MWNTs content (insert). Reproduced with permission from ref. [32].

A ductile-to-semiductile transition in the crack resistance behavior of PP/MWCNTs composites is discussed by Satapathy *et al.* [32] using an essential work of fracture approach based on a post yield fracture mechanics concept and its possible interrelation to the structural attributes studied by TEM, SEM, and WAXD. A maximum in the non-essential work of fracture is observed at 0.5 wt.% MWCNTs content, which demonstrates the enhanced resistance to crack propagation compared to pure PP, followed by a sharp decline with the increase in MWCNTs content to 1.5 wt %, which reveals a ductile-to-semiductile transition (Figure 12).

The incorporation of 0.5 wt % of MWCNTs to PP intrinsically causes α-nucleation, which favors the formation of a large number of smaller spherulites instead of larger spherulitic domains as in pure PP. Thus, the inter-spherulitic boundaries are larger in pure PP than in the nanocomposites, which facilitate the smooth advancement of the fracture once it initiates from a highly stress-localized region by the slippage of the large crystal planes of the spherulites, which causes extensive ductile yielding. On the other hand, in the nanocomposite with 0.5 wt % of MWCNTs, the excessive nucleation narrows the interspherulitic boundary and hence the extensive ductile yielding is arrested to a large extent. This causes an increase in non-essential work of fracture (*β_Wp_*) in the case of nanocomposites with 0.5 wt % of MWCNTs. Interestingly, as the MWCNTs content is further increased up to 1.5 wt. %, the nanocomposites tend to show characteristics of forming an interconnecting network. When such a network density becomes higher at higher MWCNTs concentrations, they can potentially lead to intense strain localization and hence cause partial embrittlement of the nanocomposites. The strain localization around the dense network of nanotubes ultimately causes matrix cracking because of a severe modulus mismatch between the polymer and nanotubes, and hence ductile yielding behavior is substantially reduced, *i.e.*, it becomes semiductile behavior.

**Figure 13:**
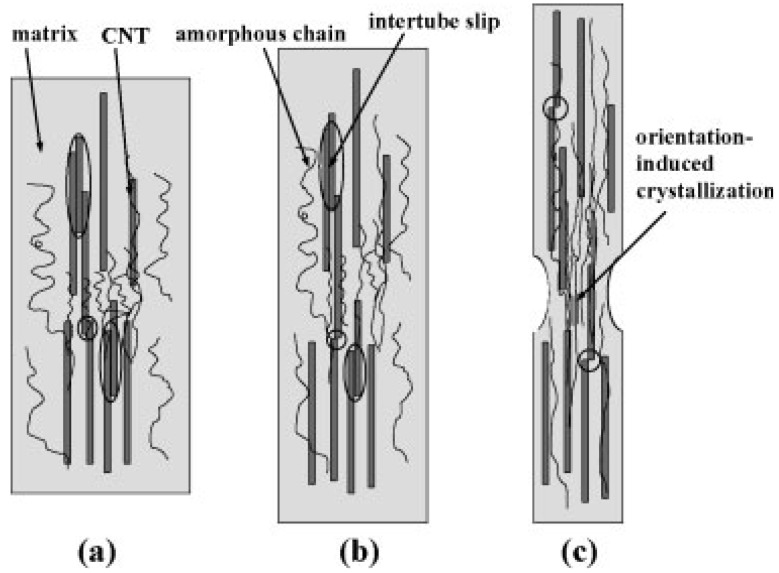
Schematic deformation model and orientation-induced crystallization of PP/CNT composite under uniaxial stress: (a) before loading, (b) under small load levels, and (c) under high load levels. Reproduced with permission from ref. [33].

Creep resistance of PP can be significantly improved by the addition of CNTs. [33]. Three possible mechanisms of load transfer were considered that could contribute to the observed enhancement of creep resistance, which are: (1) fairly good interfacial strength between MWCNTs and polymer matrix, (2) increasing immobility of amorphous regions due to nanotubes acting as restriction sites, and (3) high aspect ratio of MWCNTs. A schematic deformation model under uniaxial stress is demonstrated in Figure 13.

In fact, MWCNTs are not ideally dispersed in PP, as represented in Figure 13a with some bundles or entanglements. Consequently, the existence of intertube sliding or stick-slip may give rise to an increase in creep rate under low tensile stress, as depicted in Figure 13b. Under high tensile stress level, the large deformation of matrix may introduce high interfacial shear stress to nanotubes across good interfacial bonding. Most of the bundled tubes in the matrix will extensively slip in a short time period (compare the circled parts in Figure 13). The influence of intertube sliding becomes thereafter minor and more nanotubes are effectively transferred to sustain the external tensile load, as shown in Figure 13c with possible necking at matrix-rich parts. The significant reduction of creep rate and deformation is then achieved. The load-bearing capability is enhanced and the secondary creep stage is prolonged under high stress level.

**Figure 14:**
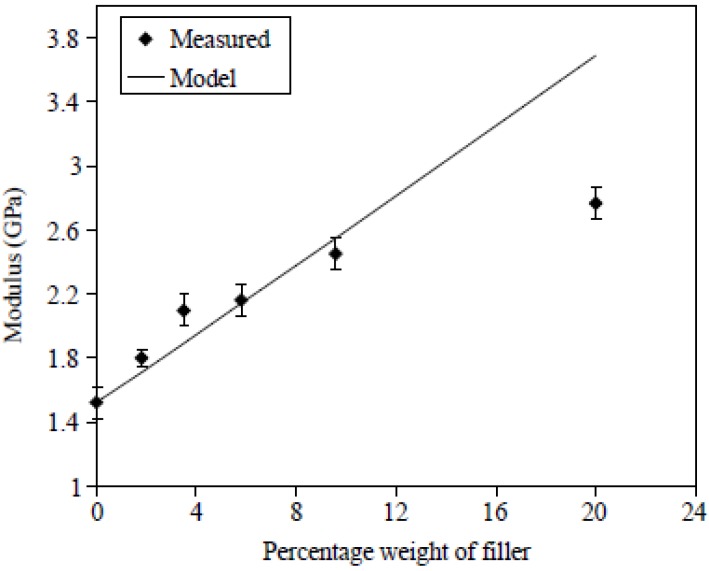
A comparison between measured and model predicted Young’s modulus of PP/CNTs composites. Reproduced with permission from ref. [34].

Tensile as well impact strength have a similar behavior in all nanocomposites containing CNTs. However, the most characteristic enhancement in mechanical properties gains the Young’s modulus. Figure 14 shows a comparison between the measured Young’s modulus of PP/CNTs composites and those predicted by the model [34].

Young’s modulus increases continuously with the increase of CNTs content, revealing the reinforcement effect of the nanotubes. It can be seen that for the sample at which the nanotube aspect ratio was actually measured, 6 wt %, the agreement between the measurement and the model is excellent. However, for samples with a lower percentage of nanotubes, the measured values fall above the predicted, while for weight percentages greater than this the predictions are lower; substantially so for the 20 wt % sample. It can be surmised that for the lower percentage samples, which spend less time in the extruder, the fiber attrition could be less and hence the modulus would be higher. For the higher weight percentage, the lower values could be due either to higher fiber attrition, or perhaps more likely to aggregation of fibers into bundles (*i.e.,* poor dispersion). A similar increase was also mentioned for nanocomposites containing higher CNTs content [29]. Composites containing 15 wt % nanotubes filling showed an improvement of the Young’s modulus of about 90%, which means in figures an increase from 410 N/mm^2^ for the pure PP to 780 N/mm^2^ for the nanofiber reinforced composite.

**Figure 15:**
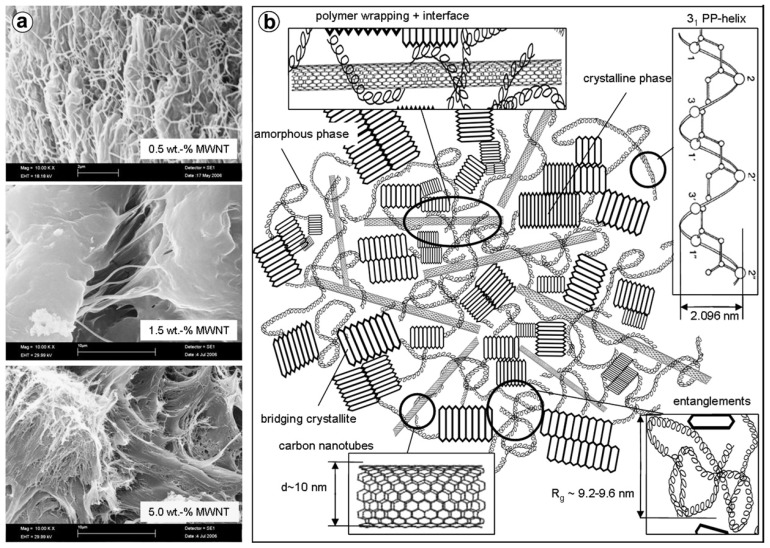
a) SEM investigation: fracture surfaces of nanocomposites with 0.5, 1.5 and 5.0 wt. % MWCNTs, and b) proposed schematic of the state of structural organization and polymer–nanotube interaction in the PP/MWCNTs nanocomposites. Reproduced with permission from ref. [19].

The study of PP/MWCNTs nanocomposites microstructure reveals the reinforcement effect of CNTs and was investigated in fractured surfaces in order to evaluate their effect on matrix deformation and the calculated mechanical properties [19]. As can be seen from the SEM images (Figure 15a) the matrix deformation is taking place with the investigation of fibrils. Furthermore, the network-density of the formed fibrils decreases in the nanocomposite with 0.5 wt % of MWCNTs when compared to pure PP, which indicates a drastic hindrance of the yielding process by MWCNTs despite plastic deformation, while still partly retaining the ductile nature of PP. With further increase of the MWCNTs content, the nanotube-rich areas become bigger and tend to show characteristics of forming an interconnecting network. When such network-density becomes higher at higher MWCNTs concentrations, they can potentially lead to intense strain localization because of hindered plastic deformation/strain of the matrix. The strain localization around the dense network of nanotubes, ultimately causes matrix cracking due to severe modulus mismatch between polymer and nanotubes and hence ductile yielding behavior is substantially reduced, *i.e.*, semi-ductile behavior. Strikingly, when the MWCNTs content was increased further to 1.5 wt %, the network density of fibrils was almost negligible/absent and instead crack-growth on the matrix surface was seen to be spanned by nanotube bridging across the cracks assisted by network of fibrils. However, the formation of a rheologically percolated network above 1 wt % of MWCNTs completely changes the matrix behavior and, thus, the crack toughness of the nanocomposite with 1.5 wt % of MWCNTs becomes a dynamic interplay of percolation and nanotube bridging leading to a semi-ductile behavior. Thus, the drastic hindrance of the ductile yielding in the polymer is controlled by the state of dispersion of the MWCNTs, the interface and MWCNTs-induced structural reorganization (nucleation).

These observations prompt first the understanding that the polymer wrapping phenomenon is being largely confined to the amorphous regions and secondly the fact that the immobilized polymer layer at the polymer–nanotube interface allows the low enthalpy mobility of the free chain ends to take place withgreater ease. The i-PP exists in a 3_1_-helix conformational arrangement with a radial dimension of 2.096 nm about its mean chain axis (c-axis). Based on these findings, a qualitative structural model about the state of the relative organization of the PP chains and nanotube has been proposed and is shown schematically in Figure 15b. The model clearly adheres to all the above detailed illustrations, though not exactly up to the scale, and fundamentally complements the understanding of the macro-scale properties correlation to nanoscale organization in the case of two component polymer based systems [19].

### 3.3. Dynamic Mechanical Analysis (DMA) of PP/CNTs nanocomposites

**Figure 16:**
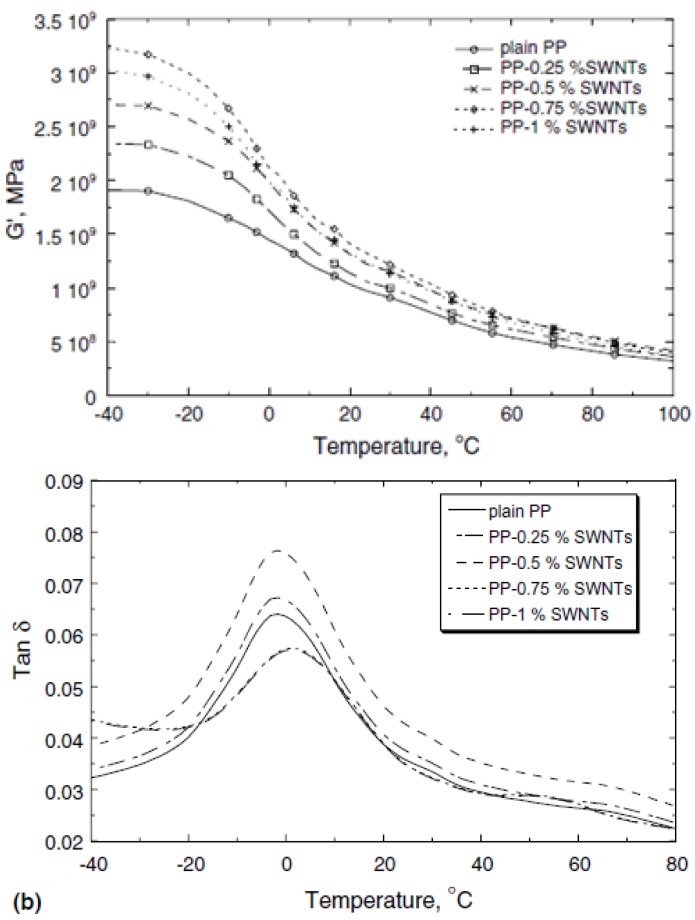
a) Storage modulus for pristine PP and its composites filled with different SWCNTs content and b) Tanδ for pristine PP and its composites as a function of SWCNTs content. Reproduced with permission from ref. [35].

The increase in mechanical properties and the reinforcement effect of CNTs can be also measured with Dynamic Mechanical Analysis (DMA). DMA of polypropylene shows three relaxations at about -80 °C (α), 8 °C (β) and 100 °C (γ), respectively. The γ peak is generally attributed to the relaxation of a few chain segments in the amorphous regions. The β-relaxation represents the glass transition, and α-relaxation is attributed to the lamellar slip and rotation in the crystalline phase. The storage modulus (G') and the mechanical loss factor (tanδ) *versus* temperature curves at 5 Hz of PP/SWCNTs are shown in Figure 16 [35]. It can be clearly observed in Figure 16a that the storage modulus of iPP is increased with the addition of SWCNTs due to their stiffening effect. However, it was found that the modulus decreases at the highest SWCNTs concentration (1 wt %). Figure 16b displays the tanδ peaks of the neat iPP and its composites filled with different SWCNTs. A displacement of the peak at higher temperatures is observed by the addition of nanotubes. Furthermore, the peak shift is more evident as higher is the nanotube percentage in the composite. This increase in the T_g_ is attributed to the reinforcing effect of SWCNTs, hindering the mobility of the chains. However, in PP/MWCNTs the glass transition temperature as determined from DMA is decreasing in the case of the nanocomposites compared to the pure iPP matrix [19,36,37]. Dynamic mechanical properties of PP/CNTs nanocomposites were found that depends also from the type of the used CNTs [38].

### 3.4. Reological behavior of PP/CNTs nanocomposites

**Figure 17:**
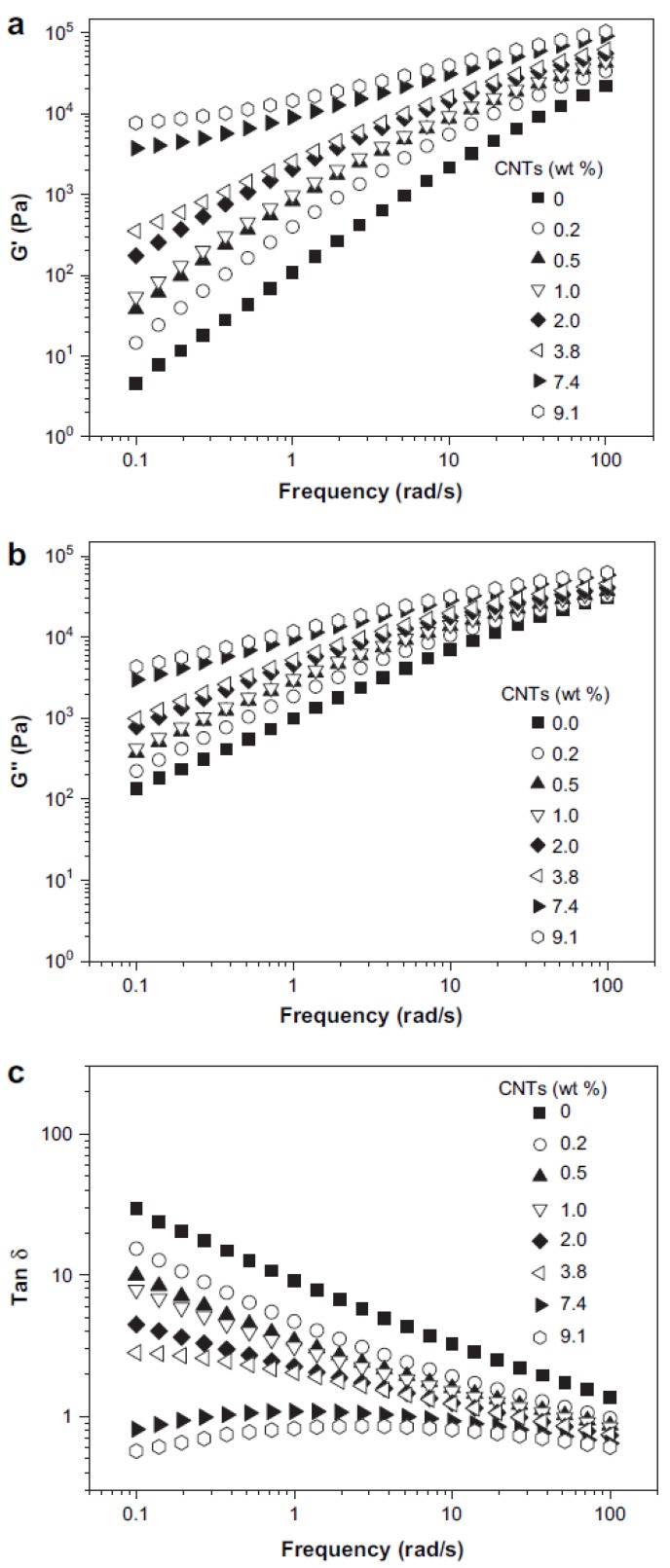
Storage modulus (G') (a), loss modulus (G") (b), and loss tangent, tanδ (c), as functions of frequency for iPP/CNTs with different CNT concentrations. Rheological measurements were performed with a strain of 2% at 200 °C. Reproduced with permission from ref. [42].

PP/CNTs at low CNTs content exhibited Newtonian behaviour, while by increasing the CNTs content (>2 wt %) non-Newtonian behavior was observed at all shear rate ranges [39]. Similar results were also mentioned from other studies [37,40,41]. Figure 17a and b shows changes of storage modulus, G', and loss modulus, G", of iPP/CNTs at different CNT concentrations with sweeping frequency, respectively. Note that the measuring temperature is 200 °C, which is above the melting point of iPP [42].

At a fixed frequency, both G' and G" increase with increasing CNTs concentration. In addition, at a fixed CNTs concentration, both G' and G" increase with increasing frequency. Moreover, it can be found that G' seems to pleateau at low frequencies for iPP/CNTs with CNTs concentrations of 7.4 wt % and 9.1 wt %. This is indicative of a transition from liquid-like to solid-like viscoelastic behavior. This non-terminal behavior at low frequencies for iPP/CNTs with high CNTs concentrations can be attributed to the formed CNTs network, which restrains long-range motions of polymer chains. It also further infers that the CNTs network may restrain diffusion of polymer chains in the undercooled melt during crystallization, leading to changes of crystallization kinetics of polymer matrix. The network formation usually is accompanied by a gelation behavior. Frequency dependence of loss tangent (tanδ) can reflect more clearly about the appearance of gelation. It has been predicted that in the pre-gel regime, tanδ monotonically decreases with increasing frequency, which is a typical behavior for a viscoelastic liquid, while in the post-gel regime, a moderate increase of tanδ at low frequencies appears with increasing frequency, indicating a dominant elastic response in the composite. Frequency dependence of tanδ for iPP/CNTs is shown in Figure 17c. It can be found that for CNTs concentrations of 7.4 wt % and 9.1 wt % moderate increases of tanδ at low frequencies do occur with increasing frequency, while for the lower CNTs concentrations monotonic decreases of tanδ with increasing frequency are always observed. Therefore, the critical gelation CNTs concentration for iPP/CNTs can be estimated to be around 7.4 wt %.

### 3.5. Effect of CNTs on PP crystallinity

CNTs cause also a serious effect on the crystallization rate of PP and this can affect most of its properties. For this reason a lot of researchers have studied extensively the effect of CNTs on PP crystallinity [30,43,44,45,46,47,48,49,50,51,52,53,54]. iPP has normally a slow crystallization rate, forming large spherulites. Nucleation can be homogeneous or heterogeneous. Homogeneous nucleation is the process in which nuclei form spontaneously in the PP melt as it cools down. This crystallization will not occur before the melt is super cooled well below the equilibrium melting temperature. On the other hand, during heterogeneous nucleation the crystallite growth is initiated by foreign bodies within the molten phase. In this case, crystallization tends to occur at higher temperatures, closer to the melting point of PP. Therefore, heterogeneous nucleation is amply used to enhance the mechanical properties and/or to provide consistent optical properties.

**Figure 18:**
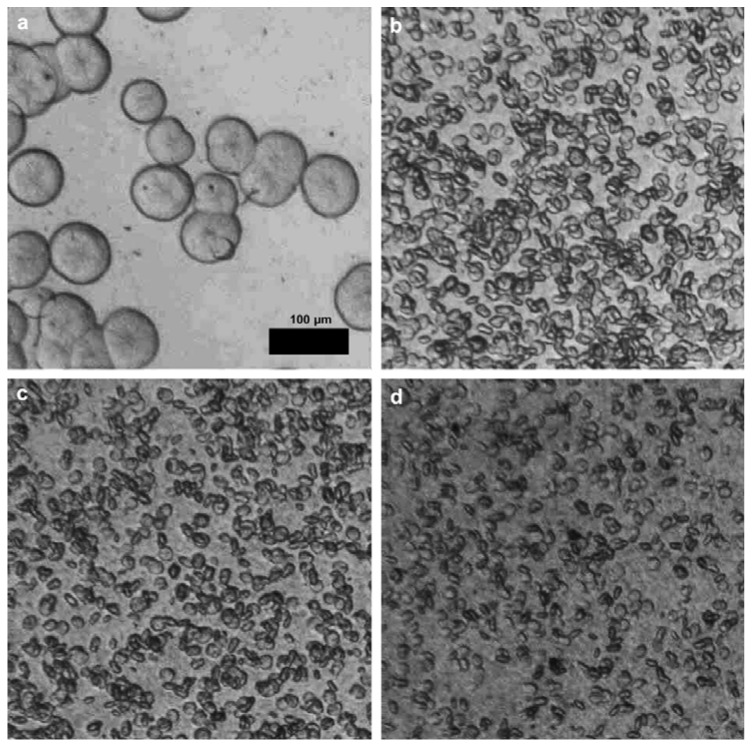
Optical micrographs of iPP/CNTs with CNTs concentrations of a) 0 wt %, b) 0.2 wt %, c) 0.5 wt % and d) 1.0 wt %, during isothermal crystallization at 136 °C for 21 min, 6 min, 6 min, and 6 min, respectively. Optical micrographs were taken in bright field. Reproduced with permission from ref. [42].

Isothermal crystallization behaviors of iPP/CNTs with CNTs concentrations less than 2.0 wt % at several selected temperatures between 136 °C and 144 °C were studied by using optical microscopy [42]. Typical optical micrographs taken at 136 °C for certain times are presented in Figure 18.

It is obviously seen that the sizes of spherulites in neat iPP are much larger than that in iPP/CNTs with CNTs concentration of 0.2 wt %. Nucleation density in the former is also obviously lower than that in the latter. Apparently, heterogeneous nucleation event is considered to occur mainly in the latter, due to nucleating effect of CNTs on primary crystallization of iPP [55,56]. It is also found that the sizes of spherulites and nucleation density of iPP/CNTs shows no obvious changes with increasing CNTs concentration from 0.2 wt % to 1.0 wt %, which infers that the nucleating effect of CNTs may saturate at least at CNTs concentration of 0.2 wt %. This implies that a further elevation of CNTs concentration does not further improve the nucleation effect [52,57].

**Figure 19:**
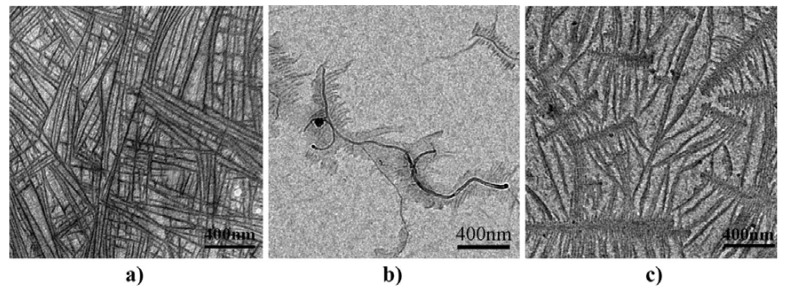
TEM bright-field images of ultrathin film samples of (a) pure iPP showing cross-hatched morphology; and transcrystalline organization of iPP around (b) MWCNTs and (c) SWCNTs (the SWCNTs are not visible). Reproduced with permission from ref. [58].

Even though a lot of published papers discuss the nucleation ability of CNTs on crystallization of semicrystalline polymers, the reasons why CNTs can be efficient nucleating agents have not been explained clearly. The authors consider that CNTs surfaces might help decrease the energy barrier for nucleation of polymer crystallization due to the evolved interactions between CNTs and PP macromolecules. For a better understanding of these interactions between CNTs and the surrounding iPP matrix, some researchers have performed crystallization experiments in ultrathin films of iPP and CNTs [58]. For this purpose, a droplet of the pure aqueous polypropylene latex dispersion or the CNTs/iPP latex dispersion was deposited on an amorphous carbon-coated TEM grid, heated to 180 °C for 5 min, and subsequently cooled to room temperature so that the iPP can crystallize. Figure 19 shows TEM bright-field images in slight defocus conditions; in contrast to stained samples, in case of phase contrast imaging as applied here, the darker lines correspond to the lamellar crystals. Again, for the neat iPP the common cross-hatched morphology is formed (Figure 19a).

As can be seen from Figure 19b, when the iPP is crystallized in contact with MWCNTs lamellar crystals are formed that grow perpendicular to the long axis of the MWCNTs. It is obvious that the iPP crystallization is nucleated at the surface of the MWCNTs, because the perpendicular orientation of the iPP lamellae follows even narrow curvatures of the MWCNTs [58].

**Figure 20:**
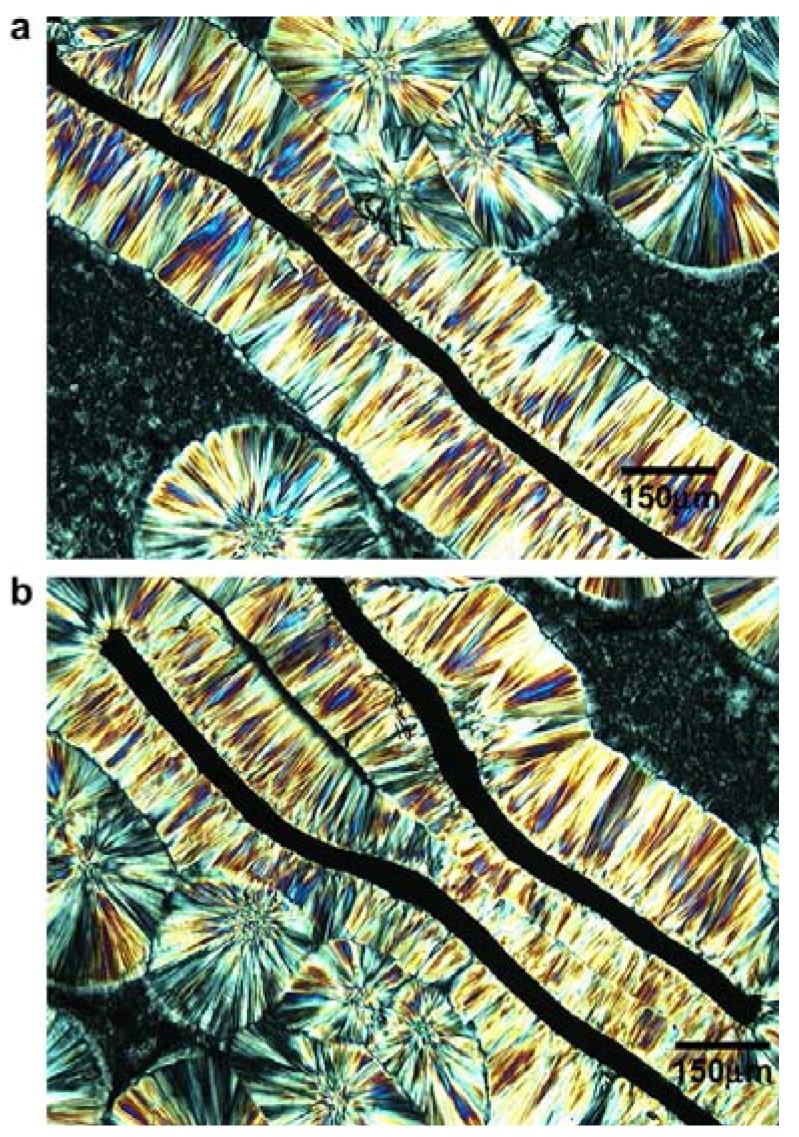
Optical micrographs of transcrystalline interphases for polypropylene surrounding the carbon fibers isothermally crystallized at 125 °C: (a) a single carbon fiber and (b) two carbon fibers. Reproduced with permission from ref. [59].

Figure 20 shows typical polypropylene morphologies around the carbon fibers in the quiescent melt at 125 °C. The images show oriented crystalline lamellae surrounding the carbon fibers. This supramolecular structure is identified as the transcrystalline interphase. Away from the carbon fibers, the polypropylene spherulites are observed. The impingement lines of the transcrystalline interphase and bulk spherulites are also observed. When two carbon fibers are close to each other, the area between two carbon fibers consists almost exclusively of transcrystalline interphases [59].

**Figure 21:**
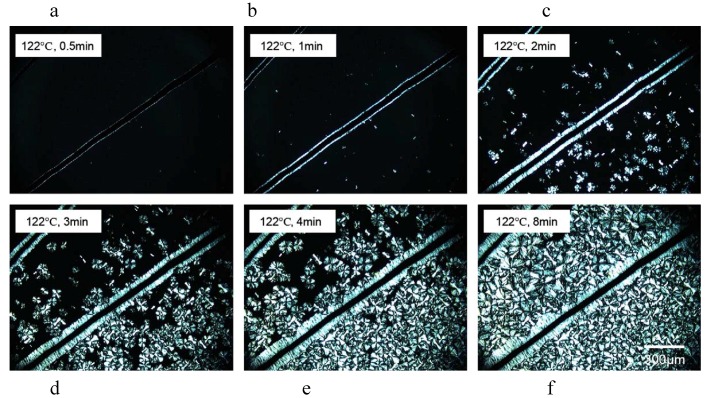
Optical micrographs under cross-polars of transcrystal developments for polypropylene surrounding the carbon fibers at 122 °C for different times: (a) 0.5 min, (b) 1 min, (c) 2 min, (d) 3 min, (e) 4 min, and (f) 8 min. Reproduced with permission from ref. [59].

PP crystallization is a dynamic process depending on the temperature (as was described before) and on the time. Figure 21 shows a series of optical images taken at 122 °C with different crystallization times. Polypropylene nucleation first occurred at the surface of the carbon fibers within 30s (Figure 21a). This uniform transcrystalline growth front indicates the high nucleating ability of the carbon fibers toward the matrix. When the crystallization time approached 1 min, the spherulite nuclei in the polypropylene matrix were also observed (Figure 21b), which grew into bulk spherulites (Figure 21c, e and f). The growth direction of transcrystals is normal to the carbon fibers axis. This growth is ultimately hindered by the impingement with bulk spherulites or by another transcrystal (Figure 21c, e and f).

**Figure 22:**
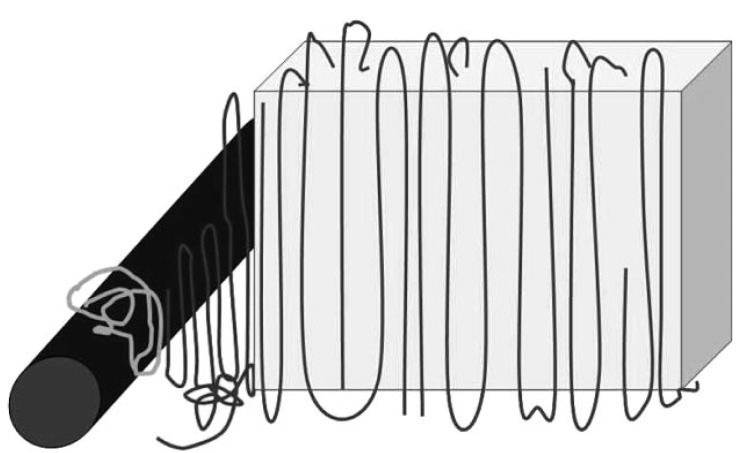
Sketch explaining the possible nucleation mechanism of iPP on the surface of a CNTs; iPP macromolecules initially are partly wrapped around the CNTs (brighter macromolecule in the sketch, dark rod represents a CNTs) and form a nuclei with crystallographic *c*-axis perpendicular to the long axis of the CNTs. Reproduced with permission from ref. [58].

The mechanism of heterogeneous nucleation is still not precisely known, it has been suggested that, apart from the dispersion of the agent, the mechanism is controlled by the polymer–nucleator interactions, and these can be chemical or physical, or even related to Van der Waals attractions. The carbon nanotubes are able to enhance the crystallization rate by increasing the nuclei sites. As a result, a raise in the crystallization temperature of the polypropylene is observed using different techniques [43,44,45,46,47,48,49,50,51]. From a recent study it was proposed that the orientation of the very first molecules, probably adsorbed at the surface of the CNTs, that induces the formation of a stable nucleus should be perpendicular rather than parallel to the long axis of the CNTs. This situation is sketched in Figure 22: iPP macromolecules are adsorbed at the surface of the CNTs in the melt and are partially wrapped around the CNTs; probably the protruding methyl groups of the iPP interact with the graphite layer of the CNT. This confinement of the macromolecules is kept during cooling of the sample from quiescent melt and causes enhanced and oriented nucleation of iPP lamellar crystals, which have their crystallographic *c*-axis tangential to the graphite shell(s) of the CNTs [58].

**Figure 23:**
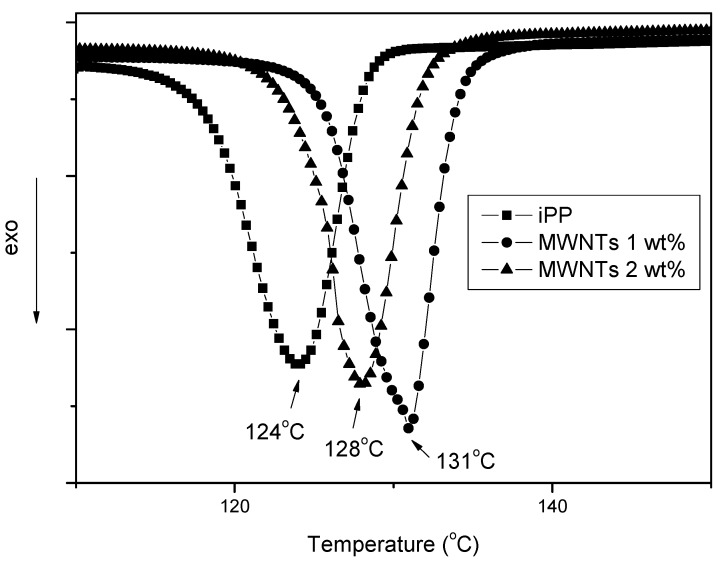
Nonisothermal crystallization curves of PP and PP/MWCNTs composites.

CNTs cannot cause only heterogeneous nucleation on PP, but also can increase the crystallization rates. The effects of CNTs on the crystallization of iPP were analyzed with nonisothermal DSC experiments on cooling. Figure 23 shows the dynamic thermograms obtained for neat PP and its composites at a cooling rate 20 °C/min. The observed dynamic crystallization behavior shows the positive effects of the nanotubes on the crystallization kinetics of PP. In particular, Figure 23 values confirm that the addition of CNTs to the polymer matrix produces an increase in *T_c_*. The relative shift of *T_c_* is quite evident at the lowest reinforcement content with a slow but continuous further increase with the CNTs concentration. These results confirm that the addition of a low concentration of nanotubes enhances the nucleation process for iPP crystallization [60].

**Figure 24:**
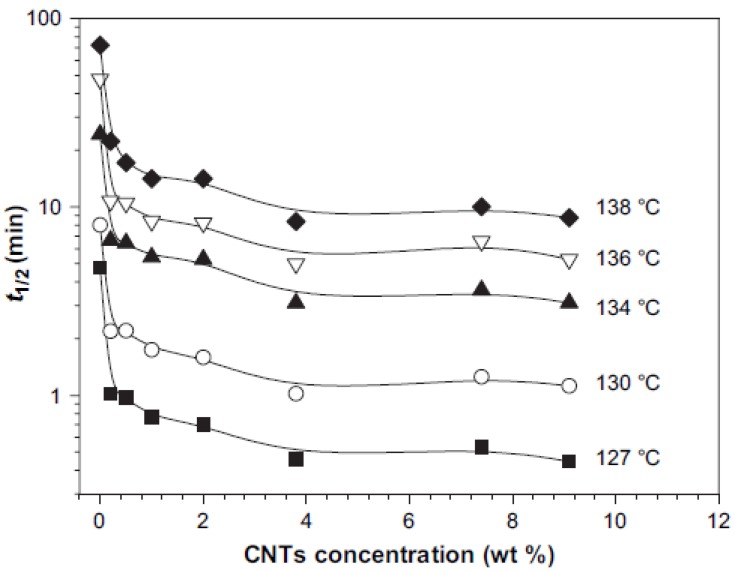
Changes of half crystallization time t_1/2_, of iPP/CNTs with different CNTs concentration at crystallization temperatures of 127 °C, 130 °C, 134 °C, 136 °C and 138 °C. Reproduced with permission from ref. [42].

To quantitatively compare isothermal crystallization kinetics of iPP/CNTs with different CNTs concentrations, the Avrami analyses on DSC data were conducted and the half crystallization time, t_1/2_, values were obtained [42]. Changes of t_1/2_ of iPP/CNTs with increasing CNTs concentration at different crystallization temperatures are shown in Figure 24. An obviously observable phenomenon is an initially rapid decrease of t_1/2_ and subsequent modest decrease of t_1/2_, followed by about constant t_1/2_, with increasing CNTs concentration at each isothermal crystallization temperature. For example, at 127 °C, addition of 0.2 wt % CNTs reduces t_1/2_ to 21% of t_1/2_ of neat iPP, additions of 0.2-3.8 wt % CNTs only reduce t_1/2_ from 21% to 10% of t_1/2_ of neat iPP, and additions of above 3.8 wt % CNTs maintain about constant 10% of t_1/2_ of neat iPP.

**Figure 25:**
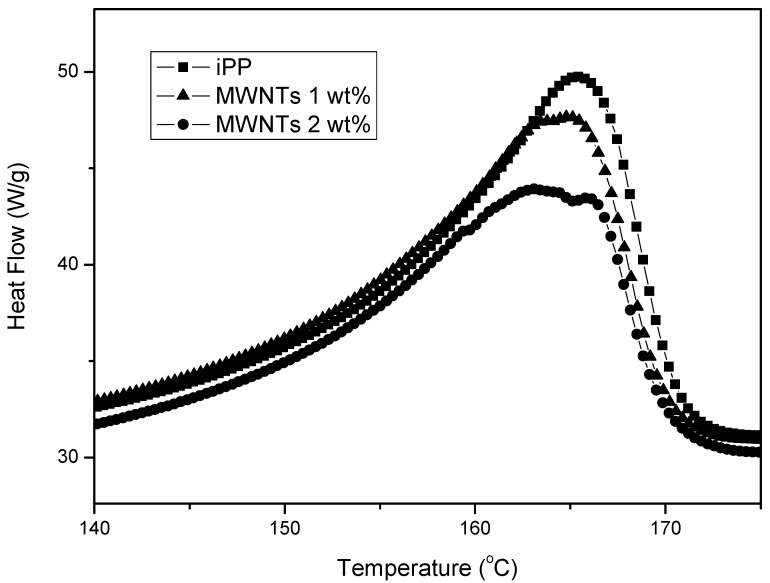
DSC thermographs for different weight percentages of nanotube loadings in PP.

The main question from the above mentioned data is if this heterogeneous nucleation as well as enhancement of crystallization rate can also increase the crystallinity of PP. Crystallinity has a major effect on the mechanical properties of polymers and hence the effect of nanoparticles on nucleation and crystallinity development is of interest. DSC thermographs of PP and PP/MWCNTs nanocomposites with different MWCNTs weight loadings are shown in Figure 25. The melting range of PP as well as its nanocomposites is 160–165 °C. A small reduction was observed in the melting point of nanocomposites compared with neat PP. Furthermore, concerning the effect of MWCNTs on iPP crystallinity, different findings were reported. As the MWCNTs loadings increased, it was reported that the percent of iPP crystallinity could remain stable [47], decrease slightly [54] or increase slightly [49].

Some studies have been done in order to determine the equilibrium melting temperature (T^o^_m_) of PP/MWCNTs nanocomposites [56]. It was found that the T^o^_m_ for neat iPP falls within the 212–215 °C range while the nanocomposites had lower T^o^_m_ than their corresponding base polymer. The presence of CNTs, and the nucleating effect associated with them (vide infra), increases the quantity of amorphous material, especially in between lamellar stacks. It may be noted that the samples prepared with functionalized carbon nanotubes had a higher T^o^_m_ than those filled with pristine MWCNTs. This is indicative of a less disrupting role of PP-g-MWCNTs, with respect to pristine MWCNTs, on the ordered and regular crystallization of the matrix.

**Figure 26:**
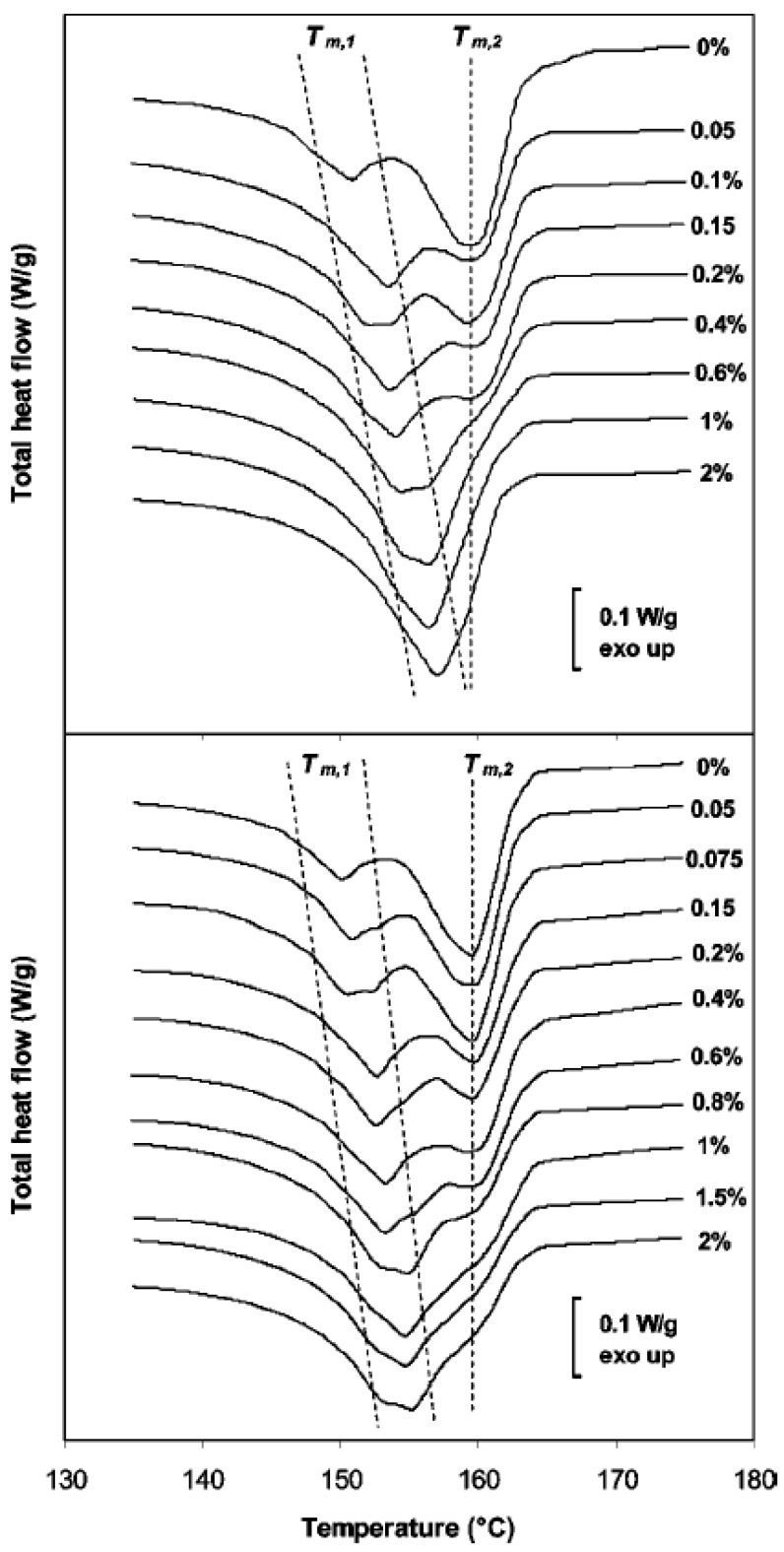
MTDSC total heat flow traces during heating at 2.5 °C/min of iPP nanocomposites containing SWCNTs (top) and MWCNTs (bottom). The dashed lines are guidelines for the eye showing the position of the melting peaks for the different systems (*T*_m,1_ marked as a broad melting region and *T*_m,2_ as a single peak). Reproduced with permission from ref. [61].

The melting behavior after nonisothermal crystallization of iPP was investigated by modulated temperature DSC (MTDSC) at a heating rate of 2.5 °C/min [61]. Figure 26 shows the melting traces for the iPP nanocomposites containing SWCNTs or MWCNTs. Clearly, the presence of CNTs has a dramatic impact on the melting behavior of the matrix material. Whereas the unfilled matrix unambiguously shows double melting behavior, the shape of the melting transition progressively evolves toward single melting with increasing SWCNTs content. Indeed, the lower melting peak *T*_m,1_ progressively shifts to higher temperature upon increasing the filler loading, until it finally almost coincides with the higher melting peak *T*_m,2_ at 2 wt % SWCNTs loading. Even though an upward shift in *T*_m,1_ can also be observed for MWCNTs composites, the effect is less pronounced than in the case of SWCNTs, with double melting behavior prevailing even at higher loading.

Multiple melting behavior is generally assumed to result from polymorphism, from the successive melting of crystal populations with distinct degrees of perfection, or from the rapid succession of melting-crystallization-melting or reorganization phenomena. The impact of fillers on the polymorphic behavior of iPP has been extensively reported over the years. Some authors reported that CNTs as well can induce crystallization of iPP in the hexagonal *β*-polymorph instead of the more common monoclinic α-form. Since both polymorphs display distinct melting temperatures, the observed changes in the shape of the melting transition in the presence of CNTs might potentially be the result of an altered balance between crystal forms simultaneously present in the sample.

**Figure 27:**
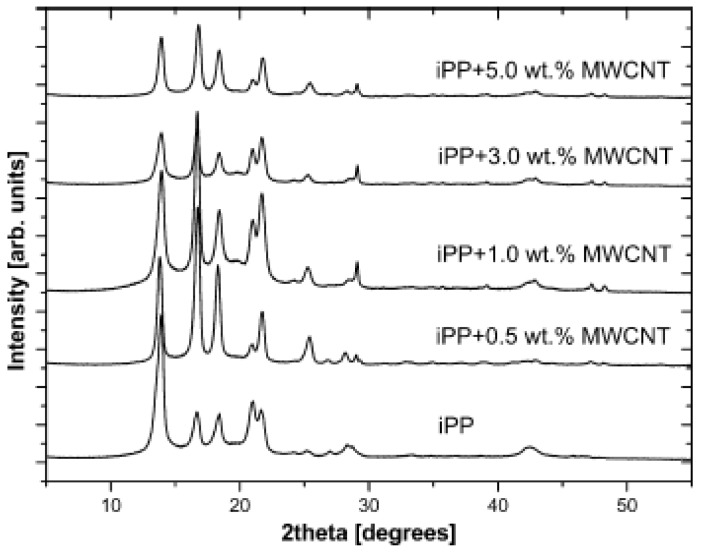
WAXD patterns of pure iPP and iPP/MWCNTs composites with differing MWCNTs contents. Reproduced with permission from ref. [13].

From the above studies with DSC, it was revealed that CNTs can have an effect on crystallization rate, melting point as well as the degree of crystallinity of PP matrix. However, it seems the CNTs can not only promote the crystallization rates and the formation of α-iPP crystals, which are the main crystal forms of PP, but can also to induce the formation of β-crystals of iPP. In DSC melting curves of pure iPP and iPP/MWCNTs composites, two obvious peaks at approximately 147 and 154 °C appeared with a MWCNTs concentration of 0.1 wt % [14]. Polypropylene in the β-crystal form melts at lower temperature than the α-crystals, so the appearance of the peaks at 147 and 154 °C can be attributed to the melting of β-crystals or smaller or imperfect α-crystals. WAXD of the iPP/MWCNTs composites confirmed the presence of β-crystals, in agreement with DSC data. Considering that the pure iPP was not able to form the β-structure crystal under identical crystallization condition, it can be concluded that the presence of MWCNTs induced the growth of the β-form crystals in iPP. This result was surprising, since most studies [13,41,45,61] suggested that MWCNTs could only promote the formation of α-iPP crystals, and no literature reported that MWCNTs could induce the formation of β-phase iPP. The characteristic crystalline reflection of α-form iPP at 2θ= 13.8° (110), 1.6° (040), 18.3° (130), and 21.5° (111,131) were observed in the curves of both pure iPP and PP/MWCNTs composites with various MWCNTs contents (Figure 27).

### 3.6. Electrical conductivity of PP/CNTs

One of the major benefits of CNTs is electrical conductivity and thus their addition into PP matrix can increase the conductivity of PP. Electrically conductive polymer composites (CPCs) based on polypropylene and conductive fillers were investigated in recent studies [13,46,47,48,49,50,51,52,53,54,55,56,57,58,59,60,61,62,63,64,65,66]. The dispersion of low concentrations (<0.5 vol %) of MWCNTs in PP, resulted in substantial decreases in the electrical surface resistivity of the derived composite material. In PP the inclusion of MWCNTs at the percolation threshold of 0.05 vol %, produced a reduction in resistivity from >10^12^ Ω/square to a value of ≈10^5^ Ω/square and higher concentrations produced further reductions to a 100 Ω/square [67]. MWCNTs form a more entangled and stable network compared to carbon black (CB) due to their large aspect ratio [68]. The aggregation of the MWCNTs, as well as CB, into sphere-like agglomerates is considered to be a main process in the formation of the conductive network in a polymer melt. The network reconstruction process during isothermal annealing is explained by re-aggregation of the conductive filler agglomerates.

**Figure 28:**
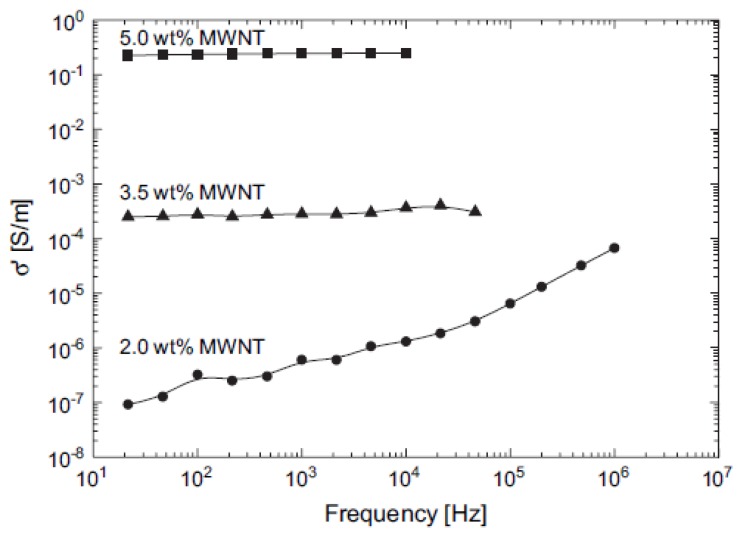
Conductivity spectra for polypropylene carbon nanotube composites with different MWCNTs contents recorded at about 120 s after the extruder was stopped. The temperature of the slit die was about 225 °C. Reproduced with permission from ref. [71].

The influence of CNTs contents on electrical properties of CNTs-reinforced PP nanocomposites was studied by many researchers. It was found that the volume resistivity of the composites was decreased with increasing the CNTs content and the electrical percolation threshold was formed between 1 and 2 wt % CNTs, which were caused by the formation of conductive chains in the composites [13,65,69,70]. It is obvious from the conductivity spectra and shape of the curves that the sample containing 2 wt % MWCNTs is close to the percolation threshold, whereas the samples with 3.5 and 5 wt % are above (Figure 28) [71]. The composite at low MWCNTs concentration is an insulator since the conductivity of the polymer matrix is taken to be negligibly small, but a “percolating” network of particles forms at higher concentrations as the randomly positioned and oriented particles begin to “overlap.” For a sample containing 2 wt % MWCNTS, the DC conductivity in the sheared melt at 220 °C is less than 10^-7^ S/m, whereas the value in the annealed melt (20 min after stopping the extruder) increased to about 1.4x10^-3^ S/m. The difference in conductivity values can be explained by a strong dependence of the ‘‘effective number of conducting bonds’’ on the arrangement of the nanotubes and clusters in the matrix as well as on the arrangement of the polymer chains in the gaps between the tubes. All these effects depend strongly on the thermo-mechanical prehistory of the sample. Thus, the DC conductivity and the static permittivity were found to increase with annealing (rest time after shear was stopped) of the melt for the sample with 2 wt % MWCNTs.

Electrical conductivity of PP/CNTs is also affected by the kind of CNTs and preparation method [17]. Microwave studies indicate conspicuous difference in the electrical properties of the iPP/MWCNTs nanocomposites as compared to the sPP/MWCNTs. Thus, the iPP/MWCNTs nanocomposites exhibit considerably higher permittivity values in comparison with the sPP/MWCNTs materials [12]. This behavior corresponds to different frequencies in microwave range. Slower growth of the sPP/MWCNTs permittivity with increasing filler content is attributed to the nanotube agglomeration.

**Figure 29:**
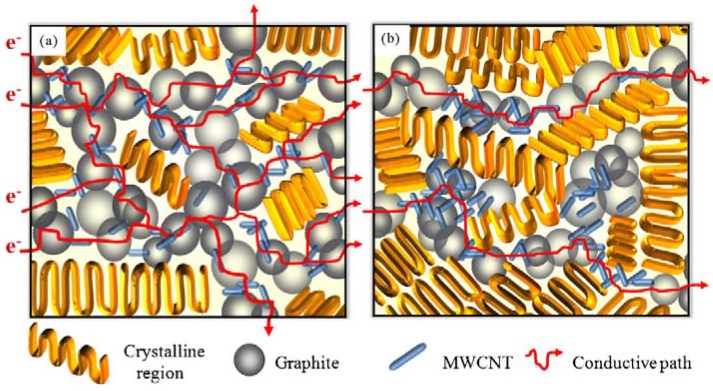
The model of conductive paths in the nanocomposite bipolar plates with (a) better dispersion of MWCNTs in LC-PP matrix (b) MWCNTs aggregation in HC-PP matrix. Reproduced with permission from ref. [73].

Electrical conductivity can be further enhanced when conductive polymers or other fillers are mixing together with MWCNTs. In a recent study, hybrid materials of conducting polyaniline (PANi) and multiwalled carbon nanotubes (CNTs) were synthesized using an *in situ* polymerization method [72]. The resulting CNTs with a thin layer of PANi coating were further compounded with polypropylene to produce electrically conductive composite material. The PANi coating was found to enhance the CNTs dispersion in PP. In a recent study, the fabrication of lightweight and high performance nanocomposite bipolar plates for the application in polymer electrode membrane fuel cells (PEMFCs) was reported [73]. The thin nanocomposite bipolar plates (thickness <1.2 mm) consisting of MWCNTs, graphite powder and PP were fabricated by compression molding. Three types of PP with different crystallinities including high crystallinity PP (HC-PP), medium crystallinity PP (MC-PP), low crystallinity PP (LC-PP) were prepared to investigate the influence of crystallinity on the dispersion of MWCNTs in PP matrix. The optimum composition of original composite bipolar plates was determined at 80 wt % graphite content and 20 wt % PP content based on the measurements of electrical and mechanical properties with various graphite contents. Results also indicate that MWCNTs was dispersed better in LC-PP than other PP owing to enough dispersed regions in nanocomposite bipolar plates. This good MWCNTs dispersion of LC-PP would cause better bulk electrical conductivity, mechanical properties and thermal stability of MWCNTs/PP nanocomposite bipolar plates. Figure 29 depicts a proposed model of conductive paths in the MWCNTs/PP nanocomposite bipolar plate with different crystallinities of PP matrices. This model was based on the presented experimental results. Electrical insulated polymers are filled with electrically conductive graphites and carbon nanotubes. The insulting zones between graphites are bridged by the MWCNTs. When voltage is applied, more continuous paths are available for the flow of electrical current due to the MWCNTs homogeneity in LC-PP matrix. Comparing these two conductive-path models, it is assumed that more electrical conductive paths were formed effectively by the better dispersed MWCNTs and graphite particles in LC-PP than that of HC-PP.

**Figure 30:**
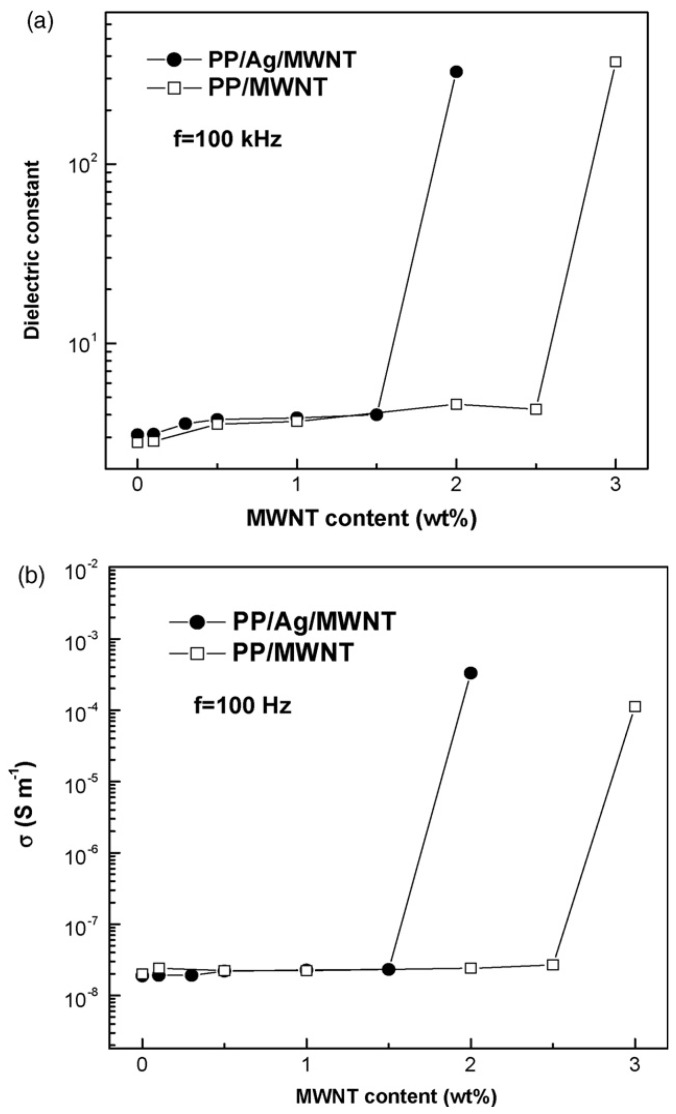
Variations of (a) dielectric constant and (b) conductivity with MWNT content for PP/MWNT and PP/Ag/MWNT nanocomposites. The nano-Ag content is fixed at 10 wt % for PP/Ag/MWNT hybrid nanocomposites. Reproduced with permission from ref. [74].

Electrical and thermal conductivity of polymers can be also increased by adding conductive metals. Silver exhibits the largest electrical and thermal conductivities among all the metals. Owing to the lack of conductive paths, PP/Ag nanocomposites exhibit poor electrical properties. In order to improve the electrical properties of PP/Ag nanocomposites, MWCNTs having large aspect ratio are compounded with PP and nano-Ag to form hybrid nanocomposites [74]. The dielectric constant and conductivity of PP/Ag/MWCNTs composites increases gradually with MWCNTs content at low filler loading. As the MWCNTs content reaches 2 wt %, the dielectric constant increases sharply by two orders of magnitude, and the conductivity by more than four orders of magnitude, exhibiting the percolation phenomenon (Figure 30).

This reveals that MWCNTs with large aspect ratio favor the formation of conductive pathways in the PP/Ag/MWCNTs hybrid nanocomposites. The nanotubes act as bridges for individual Ag particles, yielding high electrical conductivity for the ultimate composites. This clearly indicates that the Ag particles facilitate the formation of conductive pathways in the presence of MWCNTs. The opposite results were mentioned when an electrically inert additive line calcium carbonate (CaCO_3_) is used together with MWCNTs. A significant reduction in electrical resistivity of PP/MWCNTs composites was found with the addition of CaCO_3_ [75].

### 3.7. Enhancement of PP thermal stability by addition of CNTs

**Figure 31:**
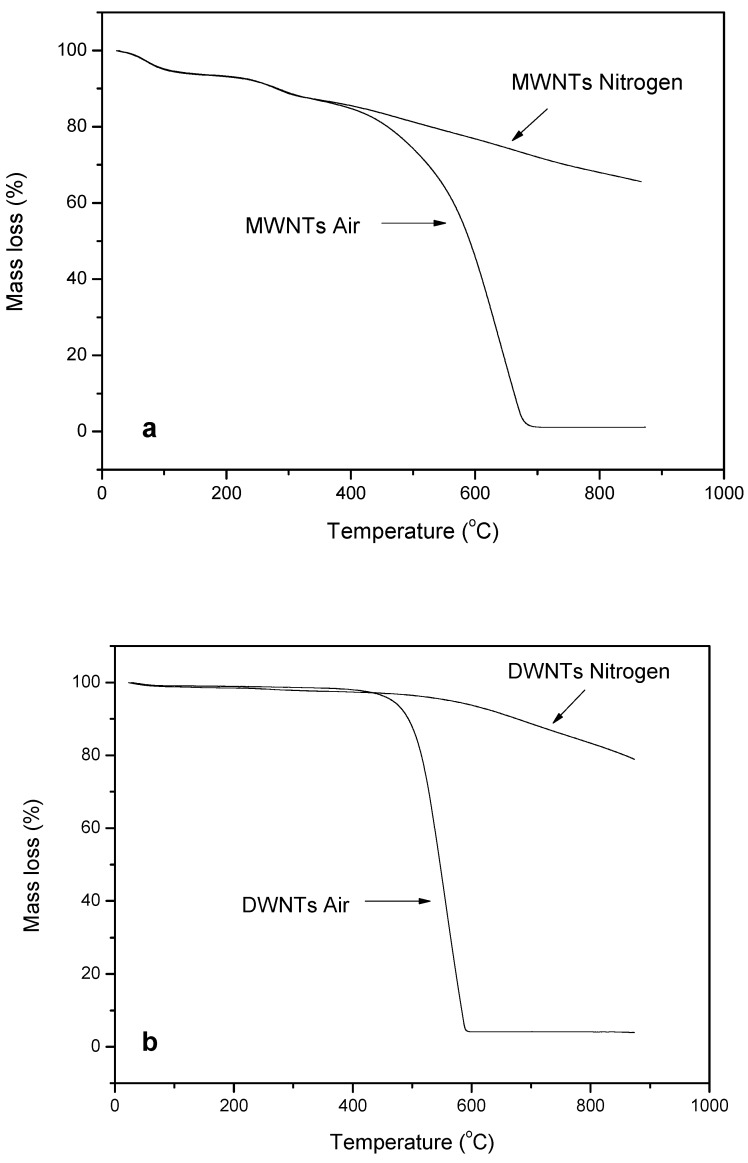
TG and DTG curves in air of MWCNTs and DWCNTs.

There are many studies concerning the thermal stability enhancement of PP reinforced with CNTs [76,77,78]. CNTs are themselves very stable materials. In air, the main mass loss of MWCNTs starts at 400 °C while the maximum decomposition rate T_max_ is found at about 600 °C. At the end of the analysis (680 °C), the degradation process is almost complete with a residue 1.5 wt %. DWCNTs start to lose mass at about 450 °C while the majority of the material degrades with a single large peak centered at about 525 °C (Figure 31). The degradation is complete at about 600 °C, leaving a 4% residue probably due to catalyst and support from synthesis. This difference can be attributed to the higher defectiveness of DWCNTs due to the synthetic process that makes them more subject to the degradative action of air with respect to MWCNTs [79]. In nitrogen, both nanotubes have similar behavior.

**Figure 32:**
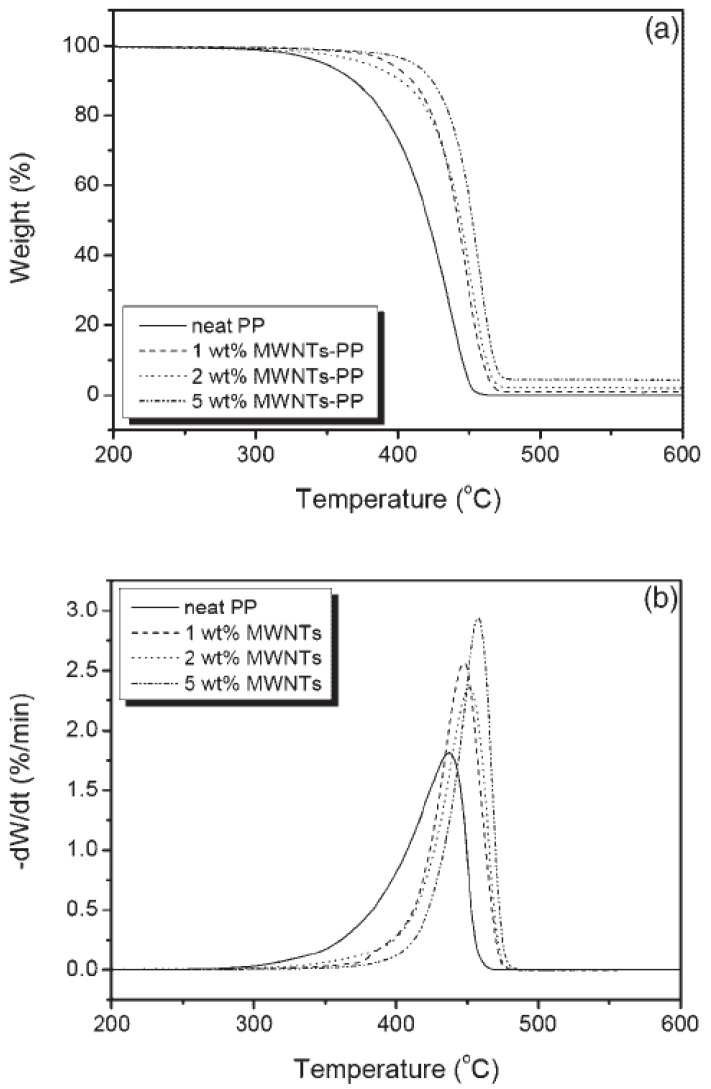
(a) TGA and (b) DTG curves of different contents of PP/MWCNTs nanocomposites at a heating rate of 10 °C/min. Reproduced with permission from ref. [78].

The thermogravimetric (TG) and derivative thermogravimetric (DTG) curves for the neat PP and PP/MWCNTs composites at a constant heating rate of 10 °C/min are shown in Figure 32(a) and (b), respectively. [54,78] It is clear that PP degrades in a single step. The step initiates at 220 °C and ends at 410 °C. The TGA curves show no significant weight loss till 250 °C. However beyond this temperature the onset of degradation was notably influenced by the nanotube loading within the polymer. The onset of thermal decomposition values increased with nanotube loading and the nanocomposite attained a maximum onset of 354 °C. Compared with the pure PP, the nanocomposites showed an increase of 16 °C with the addition of 0.5 wt % MWCNTs and 34 °C with the addition of 1.0 wt % in the onset of thermal degradation. The improvement in thermal stability can be attributed to good matrix–nanotube interaction and also due to the thermal conductivity of the nanotubes. As the nanotubes are good thermal conductors the tubes easily take up the heat that is applied to the nanocomposite fibers. The good dispersion of the nanotubes in the polymer matrix allows the spreading of heat uniformly along the fiber. Another factor that can potentially contribute to the thermal stability is the formation of a relatively uniform network-structured layer which covers the entire sample surface without any cracks or gaps forming during heating. This layer re-emits much of the incident radiation back from its hot surface, thereby reducing the heat transmitted to the PP layers below.

**Figure 33:**
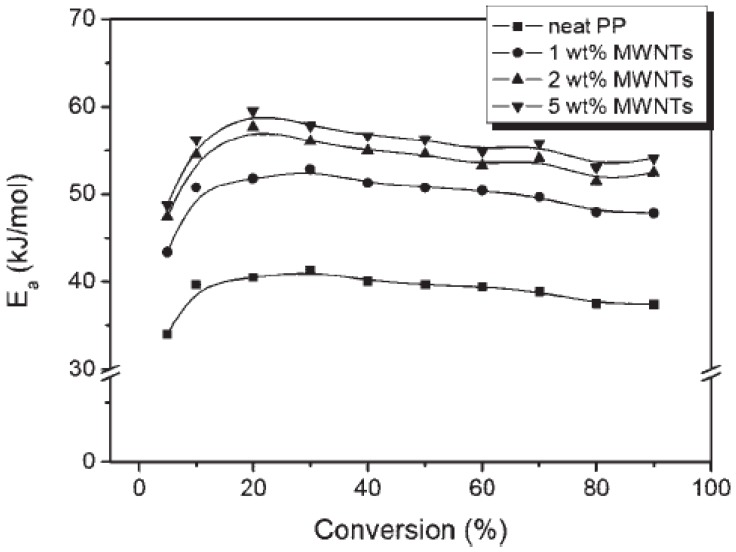
Activation energies for degradation (Ea) of different contents of MWCNTs-filled PP nanocomposites as a function of conversion using the Flynn-Wall-Ozawa method. Reproduced with permission from ref. [78].

The values of the activation energies of degradation at 50% conversion can be seen in Figure 33 [78]. There is a clear trend between the activation energy of degradation and the MWCNTs content of these samples. The activation energy values for 1 wt % PP/MWCNTs nanocomposites are 10 kJ/mol for neat PP, and increasing with increasing MWCNTs content. These results should be attributed to the increasing stability of the radicals formed during the pyrolysis process and the improving dispersion of the particles in the composites.

Thermogravimetric analyses (TGA) of PP-CNTs composites were also investigated in nitrogen and in air atmosphere in order to get a complete picture of their thermal behavior [79]. It was found that addition of DWCNTs in nitrogen increased both *T*5% and *T*_max_ of the pristine polymer with a maximum increase of 32 °C for *T*5% and of 18 °C for *T*_max_. In air, the difference between the range of temperature for composite mass loss, as compared to PP, was much higher than in nitrogen, and increased with CNTs concentration (maximum Δ*T*_max_=18 °C in nitrogen and 101 °C in air). A larger effect is observed for MWCNTs as compared to DWCNTs. The delay in *T*_max_ induced by a 3% loading of DWCNTs is in fact very similar to that induced by a 1% loading of MWCNTs. The protection action of nanotubes can be attributed to a barrier effect implying a hindered transport of polymer degradation products from the condensed to the gas phase. The shape of the mass loss curve of PP is strongly modified by the CNTs in the presence of air becoming strongly asymmetrical. At 3% MWCNTs loaded sample, the beginning of the degradation can be fixed at 320 °C, as in the case of the other samples, and the mass loss proceeds slowly up to 454 °C. When this temperature is reached an abrupt acceleration of the mass loss is observed. Such behavior can be attributed to the formation of a superficial protecting nanotube network which is destroyed when increasing the temperature up to a limit value. The protection from oxygen action is therefore extremely efficient at low temperatures but the final result is in any case a complete degradation of the polymer. The stabilizing effect of CNTs can be also ascribed to the interfacial interaction between the nanofibres and PP increasing the activation energy of degradation and to retarding the degradation at the surface of the samples owing to the better heat distribution within the heat conductive CNTs-containing samples.

### 3.8. Effect of CNTs on PP permeability

**Figure 34:**
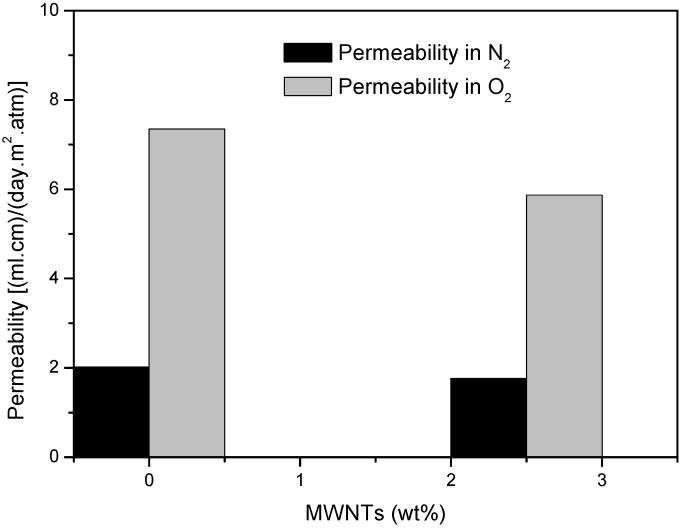
Permeability of O_2_ and N_2_ from iPP/MWCNTs nanocomposites containing 2.5 wt % MWCNTs.

Nanoparticles are well known to reduce also the permeability due to the shielding effect in PP matrices. In the case of PP/MWCNTs, the studies on gas permeability are limited [14]. Neat iPP has an O_2_ permeability of 7.35 (mL cm)/(day m^2^ atm), while this is reduced to 5.87 (mL cm)/(day m^2^ atm) after the addition of 2.5 wt % MWCNTs (Figure 34). A similar behavior is also observed for N_2_ permeability. It is well known that such nanoparticles are considered impenetrable by gas molecules. Thus, it is believed that their addition in the resin matrix would enhance its barrier properties by forcing the gas molecules to follow a more tortuous path as they diffuse through the material, retarding the progress of the phenomenon. This has been observed in many polymer/layered silicate nanocomposites as well as in iPP using fumed silica nanoparticles [80,81]. The presence of MWCNTs in the studied nanocomposites is believed to create a labyrinth, complicating the path that the gas molecules must go through in order to pass through the whole width of the film.

### 3.9. Thermal expansion of PP/CNTs nanocomposites

**Figure 35:**
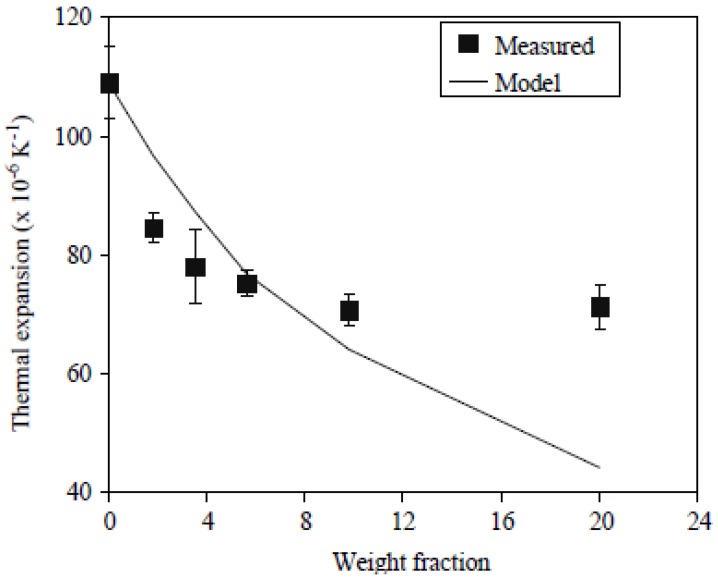
A comparison between measurements and model predictions for thermal expansion. Reproduced with permission from ref. [34].

Thermal expansion is a very important parameter for many PP products, especially since PP has been used for preparation of pipes for hot and cold water circulation. Measurements of thermal expansion behavior of the isotropic PP/CNTs composites were made on the changes in length between 15 and 25 °C, to provide an average value for a room temperature of 20 °C [34]. In Figure 35, the measurements are compared with model predictions following a similar approach to that described above for the mechanical behavior. It is interesting to note that the thermal expansion behavior mirrors exactly that for the Young’s modulus, with the predictions for the 6 wt % composite being in excellent agreement with the measurements and similar discrepancies for the other materials. For thermal expansion, the measured values for the lower weight percentages are lower than predicted and the higher weight percentages are higher, supporting the proposals above regarding the structure of these samples.

### 3.10. Flammability of PP/CNTs nanocomposites

Some brief papers involving MWCNTs were published showing significant flame retardant effectiveness of polypropylene PP/MWCNTs nanocomposites [82,83,84] even thought the residual amount of iron particles, which are used as catalyst to make the MWCNTs, can affect the thermal and thermo-oxidative stability of nanocomposites [85]. Flammability properties were measured with a cone calorimeter in air and a gasification device in a nitrogen atmosphere [83]. A significant reduction in the peak heat release rate was observed; the greatest reduction was obtained with a MWCNTs content of 1% by mass. The radiative ignition delay time of a nanocomposite having less than 2% by mass of MWCNTs was shorter than that of PP due to an increase in the radiation in-depth absorption coefficient by the addition of carbon nanotubes. The effects of residual iron particles and of defects in the MWCNTs on the heat release rate of the nanocomposite were not significant. The flame retardant performance was achieved through the formation of a relatively uniform network-structured floccule layer covering the entire sample surface without any cracks or gaps.

## 4. Effect of CNTs Functionalization on PP/CNTs Nanocomposites Properties

A good dispersion of CNTs in a polymer matrix and a strong interfacial interaction between CNTs and polymers are prerequisites to maximize the advantage of CNTs as effective reinforcing filler in polymer composites [16,10,35,77,86,87,88]. As found from almost all the reports in PP/CNTs by studying their microstructure, CNTs tend to aggregate to form bundles because of strong Van der Waal’s force, which makes the dispersion of CNTs in the polymer matrix difficult. CNTs are hydrophilic while PP is a non polar hydrophobic material. Thus, in order to achieve a finer dispersion into PP matrix CNTs should become hydrophobic due functionalization or surface coating. Consequently, techniques such as end group chemical functionalization on the CNTs and modification by grafting reactive groups on the polymer matrix have been used to improve the dispersion of CNTs in the polymer matrix [33,89,90,91,92,93,94,95].

**Figure 36:**
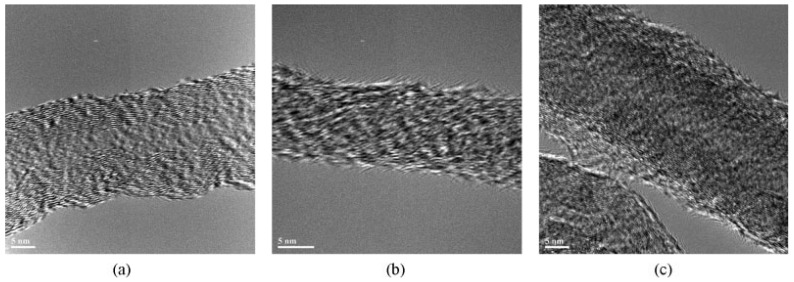
TEM images of (a) the raw MWCNTs, (b) silica-coated MWCNTs, and (c) 3-MPTS functionalized MWCNTs. Reproduced with permission from ref. [96].

Silane and alkyl groups are the most important to modify CNTs and gain a hydrophobic surface [90,96]. According to the sol-gel method, the MWCNTs functionalization can be achieved using tetraethoxysilane (TEOS). The MWCNTs were treated with TEOS:H_2_O:ethanol = 2:1:4 in a 5:1 volume ratio and the obtained dispersion was sonicated for 2 h making MWCNTs uniformly disperse and avoiding phase separation. The dispersion was maintained by stirring at room temperature overnight to allow the hydrolysis and condensation of TEOS to form silica coating on the surface of MWCNTs. After 24 h of hydrolysis, the dispersion was centrifuged three times to wash out uncoated silica from MWCNTs using ethanol to obtain silica-coated MWCNTs. CNTs can be also coated with methacryloxypropyltrimethoxysilane (3-MPTS). In this case, 1 g of silica-coated MWCNTs was dispersed in 1 L of ethanol, then the dispersion was sonicated for 30 min. 0.5 g of 3-MPTS was slowly added to 500 mL of this MWCNTs dispersion, then the mixture was refluxed for 5 h at 75 °C under constant stirring. After completion of reaction, the product was washed with deionized water and ethanol respectively five times to obtain 3-MPTS functionalized MWCNTs, which were filtrated and dried in a vacuum at 60 °C for two days. According to this procedure, the surfaces of CNTs are coated with a small layer of silica and MPTS (Figure 36).

**Figure 37:**
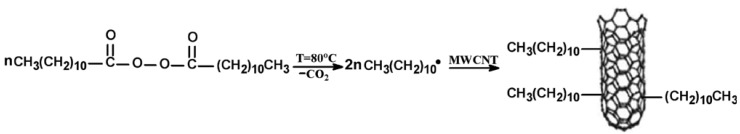
Steps for the preparation of undecyl-functionalized MWCNTs. Reproduced with permission from ref. [97].

Alkyl modified CNTs such as CNT(COOC_18_H_37_)n can also be prepared using a simple method. In the first stage, CNTs can be converted into acid form [CNT(COOH)n] via conisation in 1/3 relative volume ratio of nitric acid/sulfuric acid mixture at 40 °C and with NaOH can be converted into sodium salt form [CNT(COONa)n] [42,93]. The addition of cetyltrimethylammonium bromide (CTAB) and C_18_H_37_Br converts CNT(COONa)n into CNT(COOC_18_H_37_)n alkyl-modified CNTs. Undecyl-functionalized MWCNTs (C11-MWCNTs) were also prepared by Koval’chuk *et al.*, as described in Figure 37 [97].

**Figure 38:**
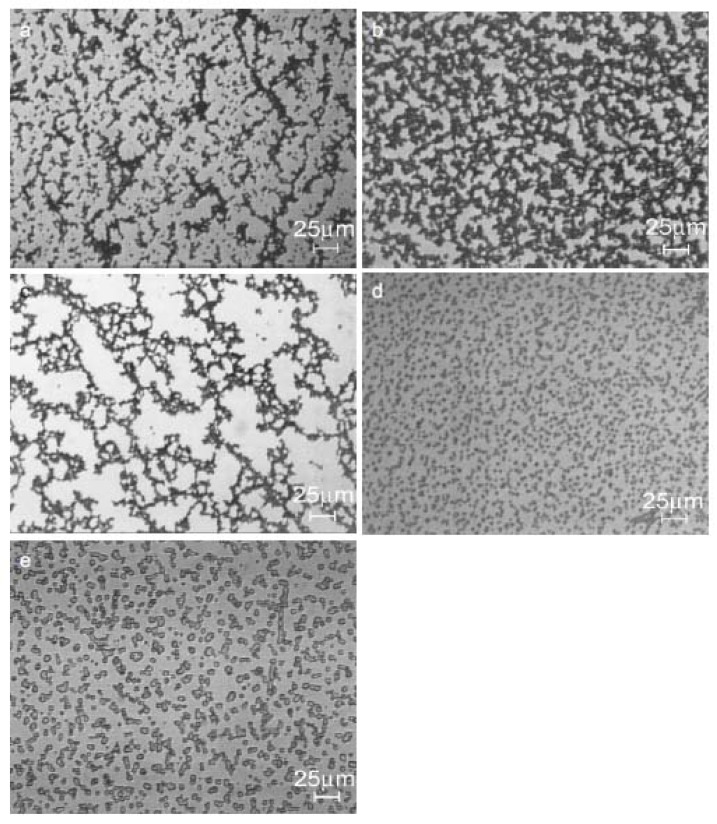
Optical microscope images of 1 wt % MWSNTs/PP composite films. The character of the CNTs dispersion was governed by surface modification via octadecylamide functionalization or surfactant adsorption: a) MWCNTs/PP; b) functionalized MWCNTAS/PP; c) MWCNTS/PP with NP-9; d) MWCNTS/PP with Triton-100, e) functionalized MWCNTS/PP with Disperbyk-163. Reproduced with permission from ref. [98].

The CNTs functionalization results in a finer dispersion of CNTs into PP matrix due to increased interfacial adhesion between the two materials. It is evident from the optical microscope images that CNTs tend to form large, non-uniform network agglomerates when dispersed in PP (Figure 38a) [98]. This is very usual in PP/CNTs nanocomposites. However, octadecyl amide-functionalized CNTs form agglomerates, which are more uniformly shaped and sized, yet still preserve their network-like-structure (Figure 38b). Except surface treatment, surfactants can be also used for finer dispersion of CNTs into PP matrix. To obtain effective steric barriers to aggregation, non-ionic surfactants with a relatively low hydrophile–liphophile balance can be applied in the preparation of MWCNTs/PP composites. While Tergitol NP-9 [poly(ethylene oxide) (9) nonylphenyl ether] surfactant, with an unbranched hydrocarbon tail, only slightly improved the untreated CNTs dispersion (Figure 38c), Triton-100, with the same head group but a branched hydrocarbon tail, generated an aggregate-free system (Figure 38d). The application of Disperbyk-163 (a commercial anionic solution of copolymers with acidic groups) was motivated by the fact that this additive is manufactured as a carbon-black dispersant that has an electrosteric stabilization effect, and was previously used as a polymer viscosity modifier. Figure 38e shows Disperbyk-163 containing functionalized MWCNTs/PP composites, and demonstrates the highly uniform distribution of the modified nanotubes within the polymer.

**Figure 39:**
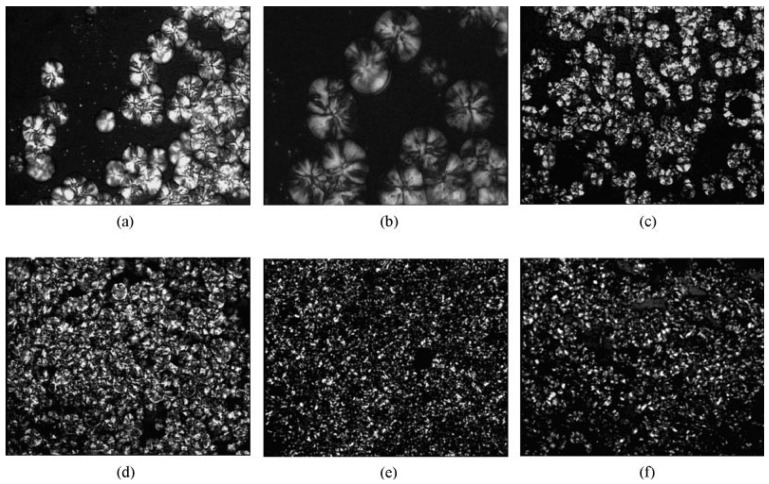
Crystalline morphology of (a) PP, (c) PP/0.5 wt % raw MWCNTs, (d) PP/0.5 wt % 3-MPTS functionalized MWCNTs, (e) PP/1 wt % raw MWCNTs, and (f) PP/1 wt % 3-MPTS functionalized MWCNTs by POM (3200 magnification); (b) PP by POM (3400 magnification). Reproduced with permission from ref. [90].

CNTs functionallization also alters the crystallization rate of PP. Figure 39 shows POM micrographs of PP and PP/MWCNTs composites that have been isothermal crystallized at 150 °C for 1 h [90]. It is clear that spherulites of pure PP are about 100 μm in diameter, larger than those of PP in PP/MWCNTs composites. The spherulites grew perfectly with the maltese cross. The addition of MWCNTs increased the amount of heterogeneous nuclei in the PP matrix, resulting in an obvious decrease in the size of the PP. When the MWCNTs content was 0.5 wt %, the spherulites grown with the maltese cross could be still observed. However, when the content of MWCNTs reached 1 wt %, only a large quantity of small crystal aggregates was visible. After the 3-MPTS functionalization of MWCNTs, for PP/0.5 wt % MWCNTs composite, the size of PP spherulites did not change, and a large quantity of spherulite crystal aggregates could be observed, indicating the 3-MPTS functionalization of MWCNTs increased the number of PP spherulites without changing the size of spherulites. When the content of MWCNTs reached 1 wt %, no difference could be found from the POM micrographs of PP/1 wt % raw MWCNTs and PP/1 wt % 3-MPTS functionalized MWCNTs composites.

**Figure 40:**
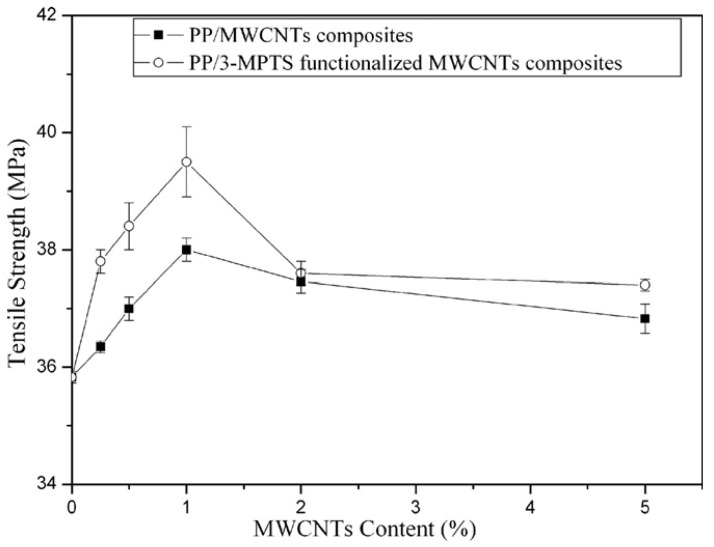
Effect of raw MWCNTs and 3-MPTS functionalized MWCNTs content on tensile strength of the PP composite. Reproduced with permission from ref. [96].

The finer dispersion of CNTs into polymer matrix further enhances the mechanical properties of the PP. Such reinforcing effect of functionalized MWCNTs on PP matrix was recently reported [96]. Comparing PP/CNTs nanocomposites with the corresponding containing functionalized MWCNTs with 3-MPTS (PP/3-MPTS) it can be seen that nanomposites with functionalized MWCNTs have higher performance. The effect of MWCNTs and 3-MPTS functionalized MWCNTs content on the tensile properties of PP composites are described in Figure 40. It can be seen that the tensile strength of PP composites increases with the increasing MWCNTs content reaching the highest values at 1 wt % MWCNTs content. The effect of 3-MPTS functionalized MWCNTs on tensile strength of PP composites is the same as raw MWCNTs. However, at the same content, the PP/3-MPTS functionalized MWCNTs composites have higher tensile strength than PP/raw MWCNTs composites.

**Figure 41:**
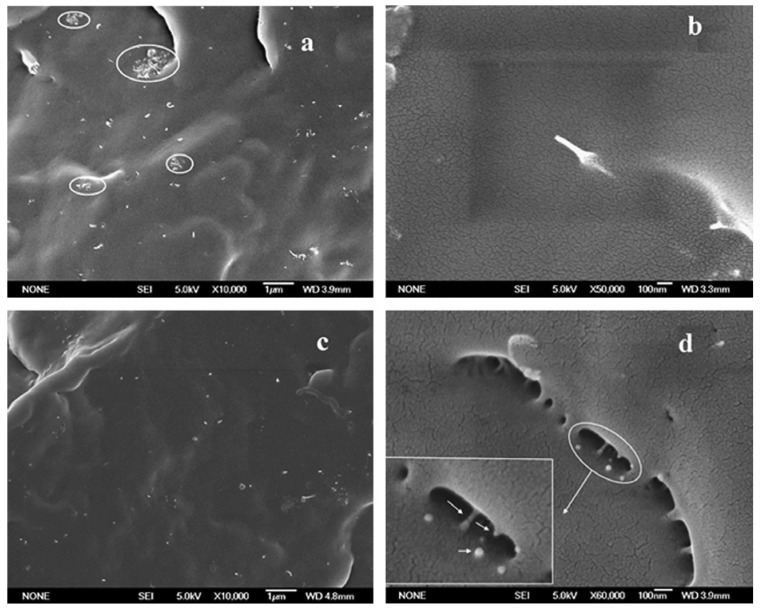
FESEM images of (a and b) PP/1 wt % raw MWCNTs; (c and d) PP/1 wt % 3-MPTS functionalized MWCNTs composites. Reproduced with permission from ref. [96].

In order to further illustrate the effect of 3-MPTS functionalization on the MWCNTs reinforced PP, FESEM was used to investigate the fracture surface of PP/MWCNTs nanocomposites [96]. The FESEM micrographs of cryogenically fractured surface for PP/1 wt % MWCNTs composite are shown in Figure 41. As seen in Figure 41a, most raw MWCNTs are well dispersed in PP matrix but some MWCNTs still exist as aggregates. 3-MPTS functionalized MWCNTs have much better dispersion than the raw MWCNTs in the composites (Figure 41c). As seen in Figure 41d, 3-MPTS functionalized MWCNTs were not simply or completely pulled out from PP matrix compared with raw MWCNTs (Figure 41b), which may be accounted from good interfacial interactions between MWCNTs and PP. For PP/3-MPTS functionalized MWCNTs composites, the interfacial interaction between MWCNTs and PP matrix could be improved through the boundary bonding between organic group chains of silane and PP matrix, and the boundary bonding could not be very strong.

**Figure 42:**
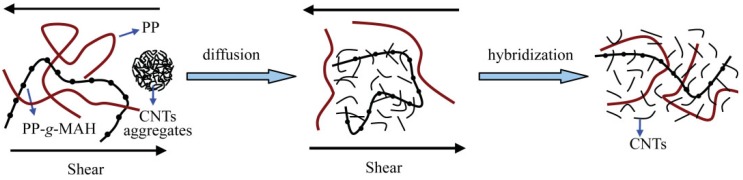
Schematic diagrams of melt compounding process for PP/CNTs nanocomposites using PP-g-MA as a compatibilizer. Reproduced with permission from ref. [102].

In most of the above described techniques functionalization of CNTs was prepared in a previous step before melt mixing with PP. However, chemical functionalization can introduce defects in nanotubes, thereby degrading their mechanical properties [99]. Thus, some researchers have used maleic anhydride grafted PP (PP-g-MA) or maleic anhydride grafted styrene–ethylene/butylene–styrene (MA-g-SEBS) as the compatibilizer to improve dispersion of the purified CNTs without further chemical modification in PP matrix [39,48,100,101]. In this case, functionalization can be achieved *in situ* during melt mixing of PP with MWCNTs. The formation of strong hydrogen bonding between hydroxyl and/or carboxyl groups of the purified CNTs and maleic anhydride groups of PP-g-MA can stabilize the morphology and enhance the interfacial interaction between PP/CNTs and finally promotes homogeneous dispersion of CNTs [100]. This confirms that PP-g-MA acts as a role of compatibilizer to improve dispersion of CNTs. The strong intermolecular van der Waals interactions among the CNTs, in combination with their high surface area and high aspect ratio as well as poor affinity to the nonpolar PP matrix, commonly causes significant agglomeration. The presence of PP-g-MA can lead to strong hydrogen bonding between carboxyl/hydroxyl groups of the CNTs and maleic anhydride groups of PP-g-MA. That noncovalent attachment, which is also called wrapping, can alter the nature of the CNT’s surface and make it more compatible with the PP because of the chemical similarity between matrix PP and grafted PP. As a result, the presence of PP-g-MA enhances the interfacial adhesions between CNTs and PP matrix and finally promotes homogeneous dispersion of CNTs, as schematically described in Figure 42 [102].

**Figure 43:**
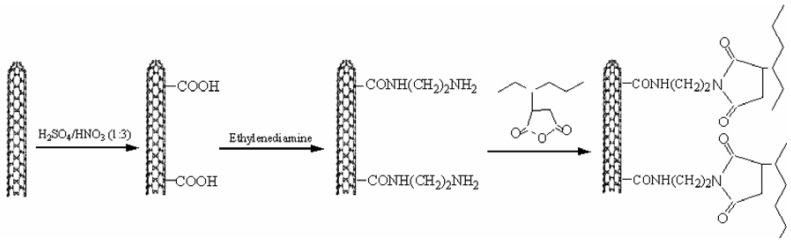
Grafting of PP onto MWCNTs. Reproduced with permission from ref. [103].

CNTs can also be covalently grafted into PP (Figure 43) with an easy procedure including oxidation of MWCNTs with a 3:1 mixture of concentrated sulfuric and nitric acid, reaction with ethylenediamine to afford MWNT–NH_2_ and finally melt blending with PP-g-MA at 220 °C [14,103].

**Figure 44:**
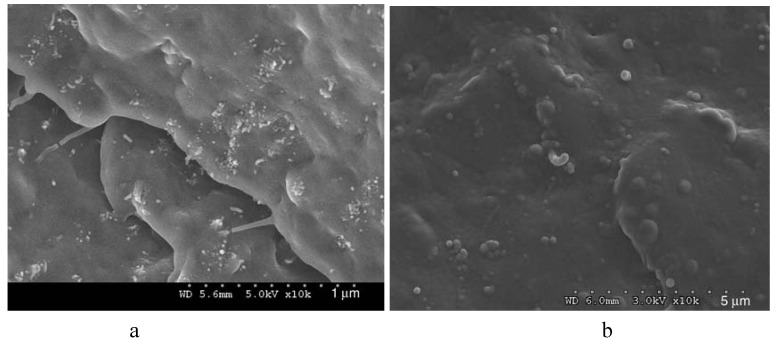
SEM images of a) CNTs-MA-PP composite and b) CNTs/PP composite. Reproduced with permission from ref. [104].

The SEM images of composites containing 3.0 wt % MWCNTs with and without PP-g-MA are shown in Figure 44 [104]. As Figure 44a shows, a large amount of self-organized MWCNTs bundles was observed on the cryofractured surface. This indicates that a significant part of the nanotubes was dispersed as nanotubes aggregates due to the imperfect mixing of the masterbatch. But in the PP/MWCNTs/PP-g-MA composites (Figure 44b), reasonably uniform dispersion and good distribution of the MWCNTs was observed with a small amount of aggregates.

From these observations, one can conclude that the added PP-g-MA promotes the dispersion of individual carbon nanotubes and limits the presence of nanotubes aggregates. Moreover, in the case of the PP/MWCNTs/PP-g-MA composite , the diameter of the MWCNTs has increased to about 150–200 nm, while in case of PP/MWCNTs diameter is about 30-40 nm. The diameter of the nanotubes was determined from different SEM observations at different places for each composite so as to be representative of the average diameter. As the nanotube used for the different composites is the same, this increase in the diameter of the nanotubes is assumed to be due to wrapping of nanotubes by PP-g-MA thanks to hydrogen bonding.

**Figure 45:**
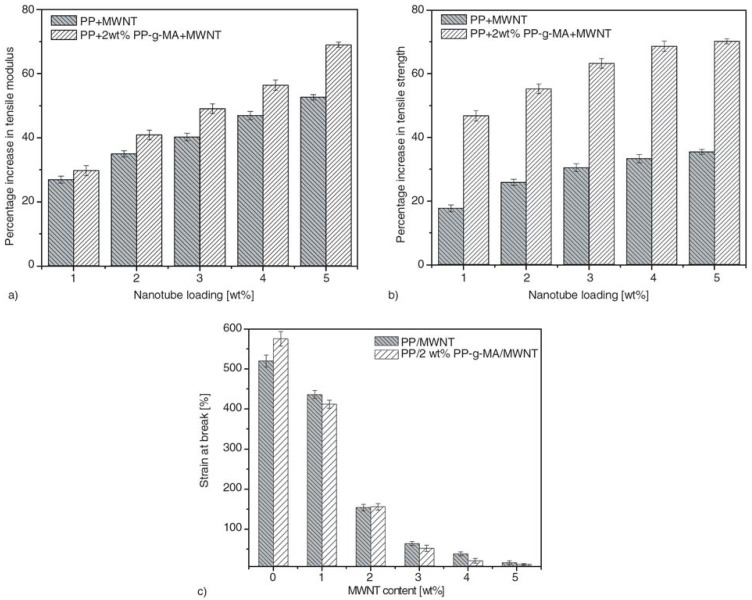
Percentage increase in tensile modulus with respect to PP and PP-g-MA as a function of MWCNTs content for the PP/MWCNTs and PP/MWCNTs/2 wt % PP-g-MA composites (a). Percentage increase in tensile strength with respect to PP and PP-g-MA as a function of MWCNTs content for the PP/MWCNTs and PP/MWCNTs/2 wt % PP-g-MA composites (b). Variation of percentage strain at break with MWCNTs content for the PP/MWCNTs and PP/MWCNTs/2 wt % PP-g-MA composites (c). Reproduced with permission from ref. [104].

This better dispersion and adhesion of CNTs with PP matrix results in a substantial enhancement of mechanical properties. However, the improvement in the mechanical properties arises from several factors. Firstly, PP-g-MWCNTs promote the crystallization of PP and the crystallites strengthen the composites. Secondly, PP-g-MWCNTs are well dispersed in the matrix, allowing a more uniform load distribution. Thirdly, the PP chains grafted onto MWCNTs enable a more efficient load transfer from the matrix to the nanotubes. The variation of tensile strength, modulus and elongation at break with MWCNTs content for the PP/MWCNTs and PP/MWCNTs/2 wt % PP-g-MA composites are shown in Figure 45 [104]. It is obvious that Young’s modulus (Figure 45a) and yield stress (Figure 45b) of PP/MWCNTs/PP-g-MA composites are higher than those of PP/MCWNTs composites, and tends to increase substantially with the MWCNTs content in composites. This was attributed to the enhanced adhesion between MWCNTs and PP matrix and the improvement of MWCNTs dispersion, which also increases the effective average aspect ratio of the nanofillers in the polymer matrix. However, in spite of the better dispersion of MWCNTs to PP matrix thanks to PP-g-MA, elongation at break decreases with increase in MWCNTs content (Figure 45c). This is probably due to the presence of few masterbatch aggregates.

**Figure 46:**
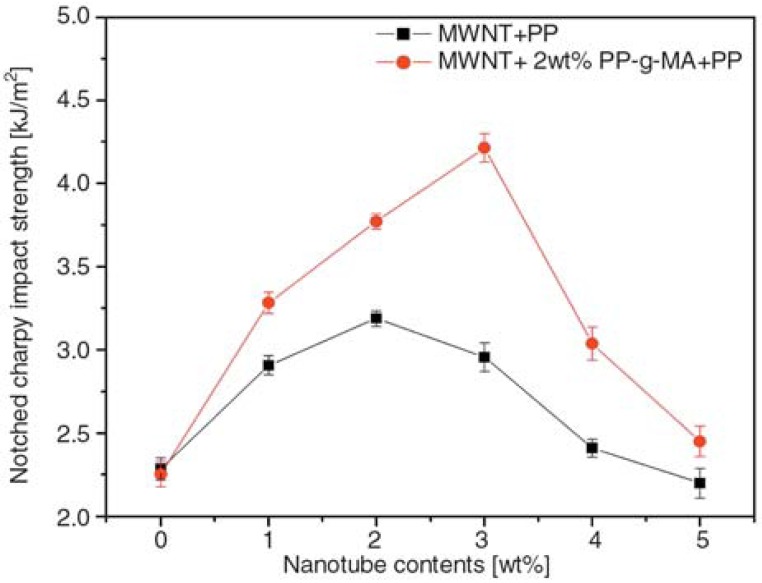
Impact properties of PP/MWCNTs (a) and PP/MWCNTs/2 wt % PP-g-MA (b) nanocomposites. Reproduced with permission from ref. [104].

Figure 46 shows the impact properties of notched samples of PP/MWCNTs and PP/MWCNTs/2 wt % PPg-MA composites. Charpy impact strength of the notched specimens slightly increased as the MWCNTs content increased up to 2 wt % for PP/MWNT nanocomposites and up to 3 wt % in case of PP/MWCNTs/2 wt % PP-g-MA nanocomposites. This increase is significantly larger for the samples with PP-g-MA. This is due to the fact that notched impact behavior is controlled to a greater extent by factors affecting the propagation of fracture initiated due to stress concentration at the notch tip. Lowering of impact energy at higher nanotube content is due to the presence fewer master-batch aggregates in the composites.

## 5. PP/CNTs Nanocomposites Prepared by *in situ* Polymerization

Bulk composites reinforced with CNTs have been investigated by melt mixing, as described before, with respect to mechanical, thermal, electrical, and other properties. However, the main problem in order to gain sufficient enhancement of most of these properties is the insufficient filler dispersion into PP matrix. Especially at high filler contents, which lead to aggregation and intercalation in turn decreasing the mechanical properties. A promising route to resolving this issue is the employment of *in situ* polymerization method [105,106,107,108,109,110,111,112,113] which involves ultrasonication for effective CNT dispersion and polymerization of monomer on nanotube surface in the present of catalysts that promotes intimate contact between polymer chains and nanotubes. By *in situ* polymerization the cocatalyst methylaluminoxane (MAO) can be absorbed or anchored on the surface of the nanofillers, changing the surface to a hydrophobic one [109]. According to this method, CNTs dispersion and subsequent propylene polymerization are accomplished in a bulk of liquid monomer [12].

Koval’chuk, *et al.*, [114] used *in situ* polymerization for the preparation of PP/CNTs composites with distinct features as follows.

**Figure 47:**
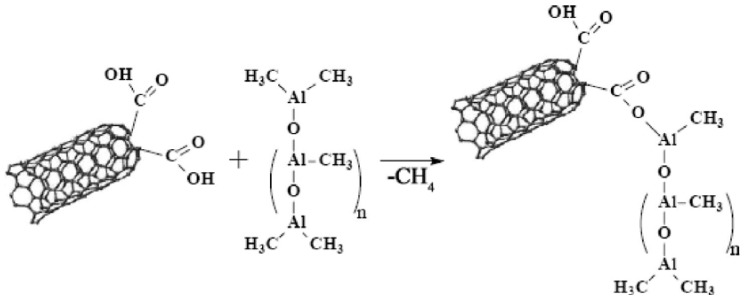
Scheme of chemical interaction between MAO and CNTs. Reproduced with permission from ref. [114].

(1) Bulk of liquid propylene is used as a medium for CNTs dispersion and subsequent polymerization reaction. Utilization of liquid monomer as a reaction medium allows obtaining of nanocomposites with high yields and high molecular weights (considering matrix polypropylene) and makes the synthesis easily scalable.
(2) CNTs dispersion via ultrasonication proceeds in the presence of the cocatalyst, methylaluminoxane (MAO). This leads to immobilization of MAO molecules on CNT surface by loose ionic interactions and, to a lesser extent, by virtue of covalent bonding (see scheme in Figure 47) to -COOH or -OH groups, which are inherent to partially oxidized CNTs.
(3) The formation of catalytic active sites is accomplished by means of heterogenization of the metallocene catalyst precursor on CNTs surface [77] owing to chemical interaction of metallocene with MAO directly during the initial stage of polymerization at lowered temperature. Covalent bonding between MAO and CNTs has considerable impact on the morphology and properties of nanocomposites; MAO molecules chemically grafted to CNTs surface form catalytic active species, yielding polypropylene chains attached directly to nanotubes.

**Figure 48:**
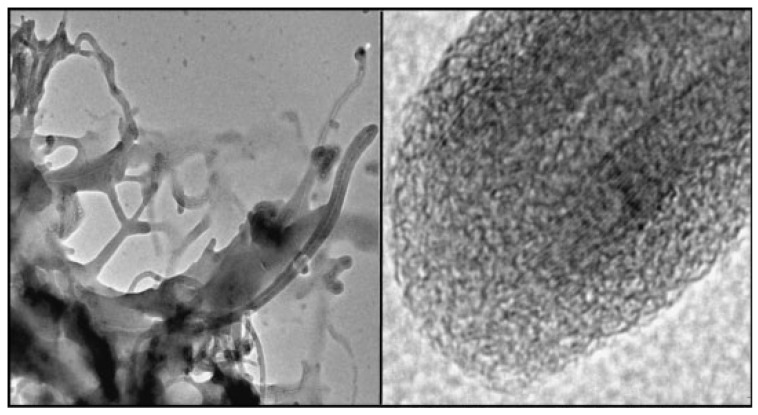
TEM image of polymer encapsulated ox. MWCNTs. Almost every nanotube is covered by a thin and homogenous *in situ* grown HMWiPP film (left). On the right: TEM images (magnification x 400,000) of an ox. MWCNTs, coated by a thin polymer film (~8 nm). Reproduced with permission from ref. [113].

Due to this reason, the polymerization of the monomer proceeds mainly in the nanotube surface. In Figure 48, CNTs encapsulated by a high molecular weight isotactic polypropylene generated by the combination of such catalyst can be seen. The detailed micrograph on the right shows the different layers (about 20) of the tube with a distance of 0.3 nm. In higher magnification it can be seen that the nanotube was covered with a high molecular weight PP (HMWiPP) film of about 8 nm thickness. Even the open cap of the MWCNTs was encapsulated too. This resulted in an excellent adhesion between tube and polymer.

**Figure 49:**
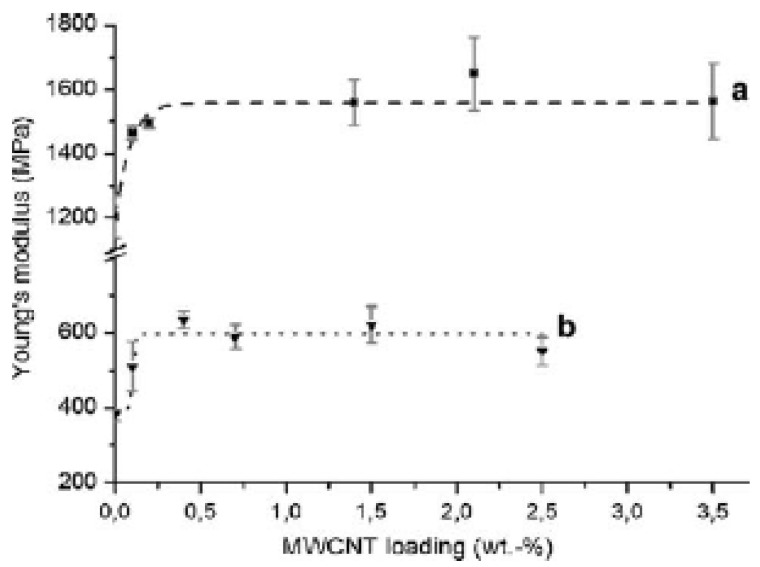
Young’s modulus of iPP/MWCNTs nanocomposites (a) and sPP/MWCNTs nanocomposites (b) as a function of MWCNTs loading. Reproduced with permission from ref. [109].

For nanocomposites prepared by *in situ* polymerization noticeable mechanical reinforcement effect is observed in the iPP and sPP nanocomposites even upon incorporating the lowest nanotube amount [12,109]. At 0.1 wt.% MWCNTs loading, Young’s modulus of iPP increases by ~22% from ~1,200 MPa to ~1,465 MPa, and modulus of sPP increases by ~34% from ~380 MPa to ~510 MPa (Figure 49). Further modulus enhancement continues up at higher filler concentrations. Ultimate Young’s modulus improvement of iPP is ~37% (from ~1,200 MPa to ~1,650 MPa) at 2.1 wt.% MWCNTs content. Subsequent moderate modulus drop at 3.5 wt.% of MWCNTs is apparently connected with the nanotube agglomeration that is inevitable at such high CNTs concentrations. For the sPP/MWCNTs composites maximal mechanical reinforcement is achieved at 0.4 wt.% nanotube loading (Young’s modulus grows by ~66% from ~380 MPa to ~635 MPa), and posterior modulus reduction at higher nanotube contents signifies considerable impact of MWCNTs aggregation that takes place in this system.

## 6. Syndiotactic PP/CNTs Nanocomposites

Syndiotactic polypropylene has been found to exhibit much superior electrical, thermal and mechanical properties compared with those of conventional isotactic and atactic polypropylene. These excellent characteristics of syndiotactic polypropylene are found to originate from lower crystallinity, much smaller spherulites, different crystal lattice and less residual catalysts than in isotactic polypropylene. However, in order to apply syndiotactic polypropylene to some applications low temperature brittleness and thermal durability should be improved. Furthermore, a drawback for its application is the relatively slow crystallization rate [115].

**Figure 50:**
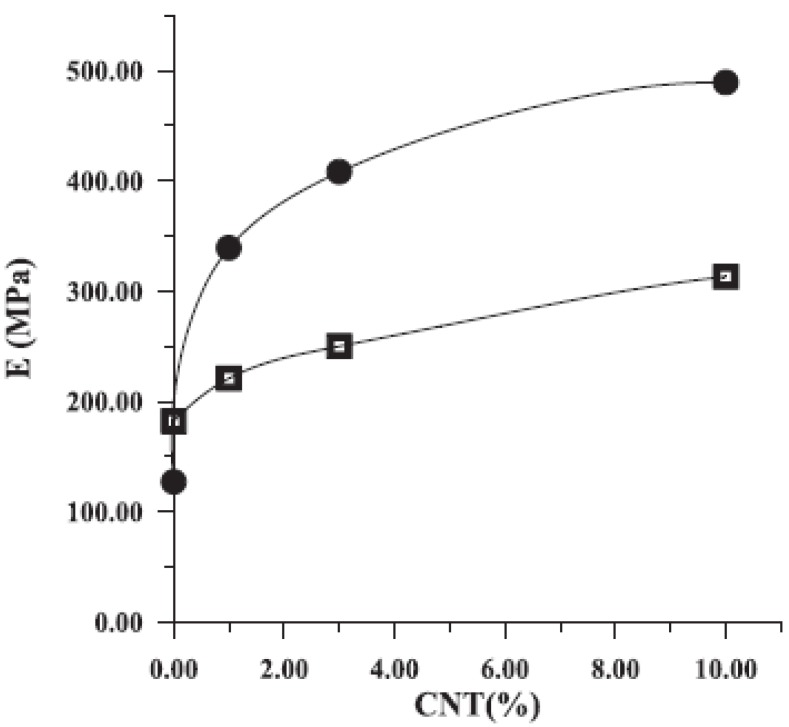
The elastic moduli for samples quenched at 25 °C (●) and at 100 °C (◘). Reproduced with permission from ref. [116].

The addition of CNTs was reported that can further enhance the mechanical properties of sPP [116,117]. Figure 50 shows the elastic modulus of all the samples of sPP, as a function of the CNT content.

As can be seen, the modulus increases with increasing CNTs content. The enhancement of the mechanical properties of the composites relies on many factors, the most important being: the morphology of the polymeric matrix, in terms of crystallinity and crystal dimensions, the good dispersion of the nanotubes into the polymeric matrix and the strong interfacial adhesion between the phases, leading to a high degree of load transfer between the matrix and the nanotubes. Furthermore, the modulus of the sample crystallized at 25 °C (25sPPCN0) is lower than that of sample crystallized at 100 °C (100sPPCN0), due to the higher crystallinity degree of the latter sample, which seems to be the predominant factor. However, an inversion is observed for the nanocomposites. The increase of the modulus with CNTs content is more relevant for the nanocomposites quenched at 25 °C, while a less dramatic effect is observed for the samples quenched at 100 °C. This result must be correlated with the morphology of the amorphous phase in both samples. The carbon nanotubes are dispersed in this phase, and their behavior depends both on their properties (dispersion and interaction) and on the amorphous phase interconnection with the crystalline phase. In the samples crystallized at lower temperature (25 °C) the crystals are initially smaller and the amorphous phase is better dispersed between the small crystals, forming a more interconnected matrix.

**Figure 51:**
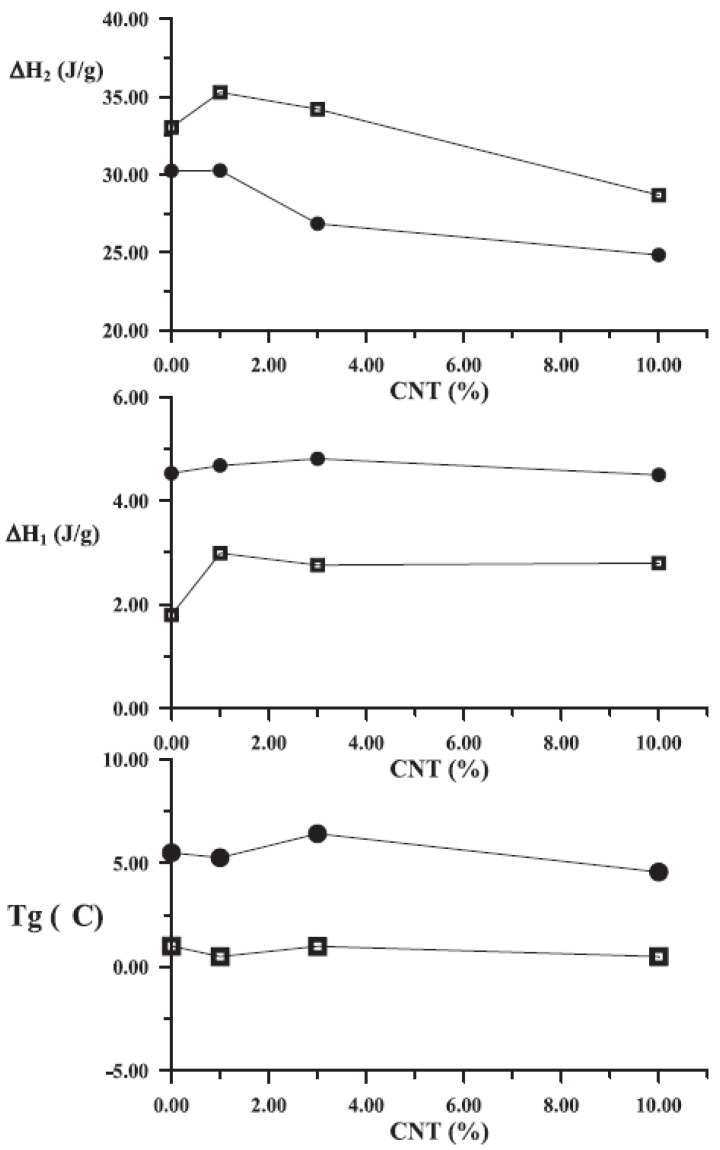
The glass transition temperature (Tg (°C)), the melting enthalpy of the mesophase (ΔH_1_ (J g^-1^)), the melting enthalpy of the crystalline phase (ΔH_2_ (J g^-1^)) for samples quenched at 25 °C (●) and at 100 °C (□). Reproduced with permission from ref. [116].

Thermal properties of sPP are also affected from the addition of CNTs (Figure 51). The main result regarding the glass transition temperature is that, besides small variations on increasing the CNTs content, the glass temperature of 25sPP samples is always consistently higher than that the T_g_ of the 100sPP samples. This indicates that the higher temperature of crystallization of 100 °C influences the amorphous phase, making the chains in this phase more relaxed with respect to the samples crystallized at 25 °C. Also the melting enthalpy of the mesophase is consistently higher in samples crystallized at 25 °C, and it increases on increasing the CNTs content [116].

**Figure 52:**
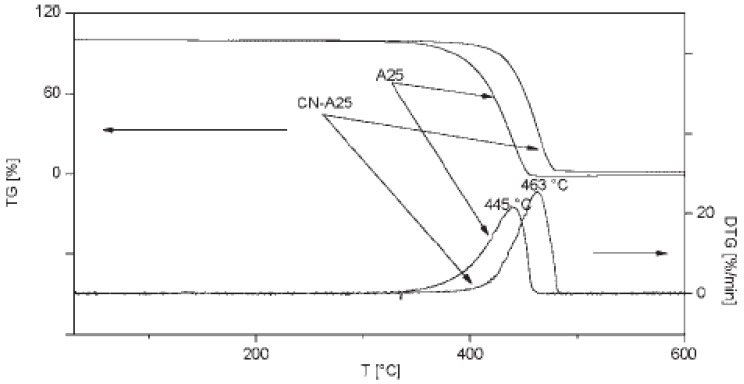
TG-DTG analysis in nitrogen of sPP/CNTs nanocomposites A25; CNA25. Reproduced with permission from ref. [117].

Carbon nanotubes also improve the thermal stability of sPP in nitrogen as well as in air, although the complex behavior of the degradation in air was interestingly found to depend on the structural organization and morphology of the composite samples [117]. The thermal degradation of sPP is caused by the scission of C–C bonds followed by hydrogen transfer at the site of scission. Samples containing CNTs (CN-A25) also degrades in a one-loss step, while both the onset of the degradation temperature and the peak temperature of the derivative weight loss (DTG) are shifted to higher values by about 20^ο^C (Figure 52). This result, which is similar to that reported for isotactic polypropylene, could be explained by a barrier effect of carbon nanotubes, which hinders the diffusion of the degraded products from the bulk of the polymer to the gas phase.

**Figure 53:**
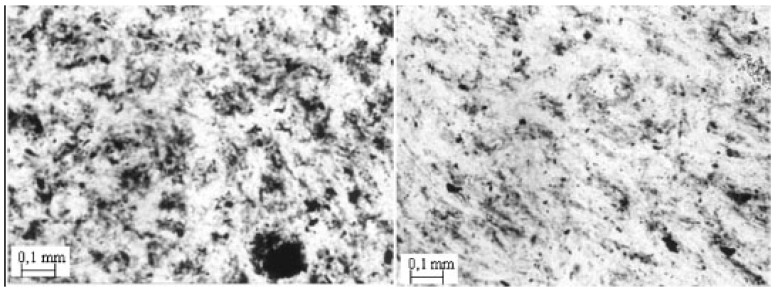
Micrographs of sPP/MWCNTs nanocomposites prepared with prereaction after sonication of the nanotubes with different amplitudes (left: 10%, right: 30%). Reproduced with permission from ref. [107].

Syntiotactic PP nanocomposites with MWCNTs were also prepared by *in situ* polymerization. However, different pre-treatments before the polymerization were necessary to achieve a homogeneous distribution of MWCNTs [107]. Preliminary experiments showed that MWCNTs stay aggregated if not treated with ultrasound prior to the polymerization. The effect of different ultrasonic amplitudes (which are a measure of the energy input) and various sonication times was investigated. Higher amplitude led to a better dispersion. Figure 53 shows microscopic photographs of sPP/MWCNTs nanocomposites prepared without pre reaction after treatment of the MWCNTs with ultrasonic amplitude of 10 or 30%. It can be seen that a higher amplitude leads to a slightly better dispersion, which is still not satisfactory. To achieve a more homogeneous distribution, the MWCNTs were stirred with MAO solution overnight after sonication (with pre-reaction). The results showed that the pre-reaction with MAO has led to a much more homogeneous dispersion of the fillers in the matrix. During the pre-reaction with MAO, hydroxyl groups on the nanotube surface can react with the MAO by the formation of covalent oxygen-aluminum bonds. The metallocene is then heterogenized indirectly on the MWCNTs. This should lead to polymer growth directly on the filler surface to resulting in a better dispersion compared to nanocomposites prepared without pre-reaction.

## 7. Polypropylene Polymer Blends with CNTs

**Figure 54:**
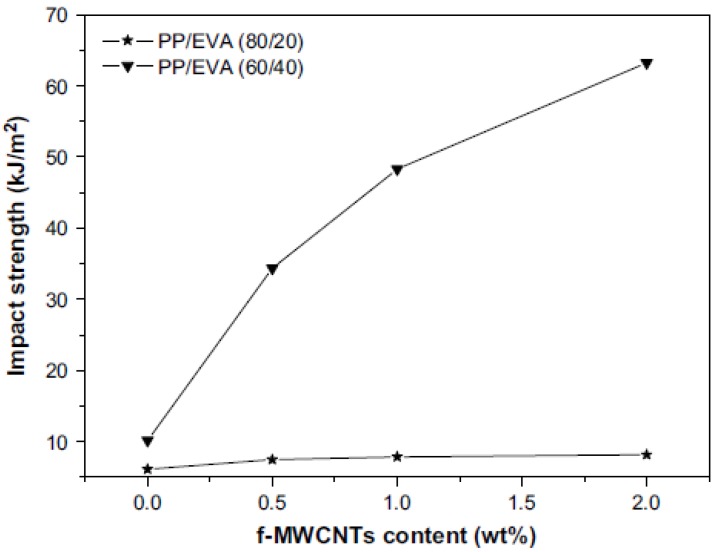
Notched Izod impact strength of PP/EVA blends with different contents of functionalized MWCNTs (f-MWCNTs). Reproduced with permission from ref. [122].

Besides the application of CNTs in single polymer matrices, other researchers have shown that CNTs also exhibit apparent roles in influencing the morphologies, mechanical and electrical properties of PP polymer blends [118,119,120]. Most of these blends are immiscible and show poor mechanical properties because of the weak interface between the two phases. The toughening of PP by rubbers, particularly ethylene–propylene copolymers and terpolymers (EPM and EPDM, respectively), was found to be highly effective and can be further enhanced by the addition of carbon nanotubes [121]. For example, although poly(ethylene-co-vinyl acetate) (EVA) has been proved to improve the fracture toughness of PP, the toughening effect is still inconspicuous. The addition of a few amounts of MWCNTs induces the change of fracture toughness of composites in different degrees. The improvement of impact strength is greatly dependent of the contents of EVA and MWCNTs. For PP/EVA 80/20 w/w, the impact strength increases slightly with the increasing content of MWCNTs, indicating that MWCNTs do not influence the fracture toughness of such blend significantly (Figure 54). However, for PP/EVA 60/40 w/w, the impact strength increases greatly with increasing content of MWCNTs. This may provide a simple but efficient way to improve the mechanical properties of such immiscible polyolefin blends and this improvement depends from the morphological characteristics of the blends [122].

**Figure 55:**
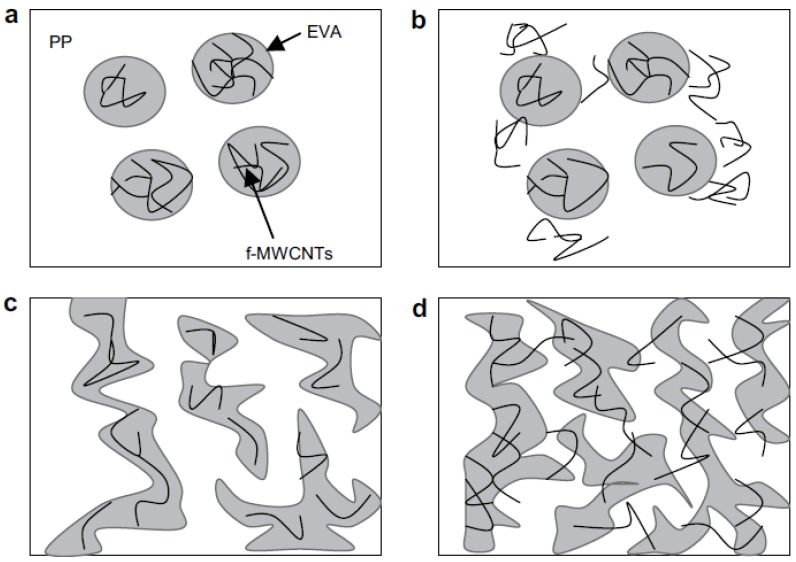
Schematic representations of the dispersion states of f-MWCNTs in PP/EVA blends. (a): f-MWCNTs tend to aggregate in dispersed EVA phase in PP/EVA (80/20) at low load; (b): f-MWCNTs migrate to PP phase and form a local ‘‘single-network structure’’ at high load; (c): f-MWCNTs exhibit good dispersion in continuous phase of EVA in PP/EVA (60/40) at low load and (d): f-MWCNTs form the network structure in the whole blend. Reproduced with permission from ref. [123].

For PP/EVA 80/20 w/w with low MWCNTs content, MWCNTs tend to form clusters, which are limited in the EVA phase (Figure 55a); at high f-MWCNTs content, these clusters migrate to PP phase, possibly inducing the local network structure or aggregation of MWCNTs around EVA particles (Figure 55b). For PP/EVA 60/40 w/w, MWCNTs exhibit good dispersion in EVA phase at low load due to the largely increased EVA phase and the good interaction between EVA and MWCNTs (Figure 55c). At high load, MWCNTs form the network structure in EVA phase. Furthermore, some MWCNTs tend to migrate to PP phase, possibly inducing some MWCNTs span the two phases, leading to the bridge effect for PP and EVA (Figure 55d). Khare *et al.* [123] revealed also a co-continuous structure and a refinement in the morphology of polypropylene/acrylonitrile–butadiene–styrene (PP/ABS) blends in the presence of CNTs. Furthermore, lower electrical percolation threshold was observed in continuous PP/ABS blends with CNTs.

## 8. Summary and Conclusions

Carbon nanotubes (CNTs) show promise to revolutionize several fields in material science and are suggested to pave the way into nanotechnology. The discovery of carbon nanotubes and carbon nanotube based materials has inspired scientists for a range of potential applications due to their extraordinary mechanical, electrical, and optical properties. Isotactic polypropylene (iPP) is a thermoplastic polymer widely used in many fields and its applications are increasing last years. It has been reported that carbon nanotubes in iPP produce the following improvements in properties:

Young’s modulus, tensile strength, ultimate strain and toughness of iPP can be improved since neat CNTs have higher mechanical properties than neat iPP.

CNTs/PP nanocomposites exhibit superior thermal stability compared to polyolefin materials.

Electrical and thermal conductivity are also enhanced by the addition of CNTs creating a conductive network in the polymer matrix.

It has been demonstrated that the incorporation of nanotubes affects the crystalline behavior and structure of the iPP matrix. In particular, CNTs accelerate the heterogeneous nucleation and crystal growth mechanisms of iPP, this effect being more noticeable at the lower filler content analyzed. From optical microscopy studies, it was found that the iPP spherulites decreased in size when CNTs was introduced into the polymer. The nonisothermal crystallization rate increases up to 2-4 wt % CNTs and then decreases slightly or remains almost constant at higher CNTs content. The decrease of the nucleation efficiency at high filler concentration is not due to a change in the crystallization mechanism, but to an aggregation of the filler particles at high concentration, which leads to a decrease of the number of heterogeneous nuclei.

However all these improvements depend on the amount of CNTs added in polymer matrix and mainly on the prepared microstructure. One of the advantages of CNTs as reinforcement agents is their large surface area, which can induce better adhesion with the polymeric matrix, which is an important factor for an effective improvement of the composite properties. However, CNTs tend to aggregate into bundles and hence they are difficult to be dispersed homogeneously in polymer matrices. For this reason mechanical properties are reducing after an added amount 2-3 wt % of CNTs.

Furthermore, the polymer/CNTs interfacial adhesion is weak, preventing an efficient load transfer from the polymer matrix to CNTs. As a result of poor dispersion and inefficient load transfer, the mechanical properties of polymer/CNTs composites are often not as good as anticipated. Appropriate surface treatment of nanotubes will result into improved fiber to matrix interaction. This in turn will enhance the dispersion and wetting properties of the nanotubes leading to further property improvement of composite materials. CNTs can chemically modified by grafting alkyl chains to improve compatibility between CNTs and iPP and to enhance dispersion of CNTs in iPP matrix. CNTs can also be grafted with functional groups to facilitate better dispersion of nanotubes within iPP.

Nanocomposites based on iPP and different types of CNTs could successfully be also prepared by *in situ* polymerization of propylene with a metallocene/methylaluminoxane catalyst system. In this case mechanical properties can be further enhanced due to increased matrix adhesion.

## References

[1] Balow M.J., Karian H.G. (2003). Handbook of Polypropylene and Polypropylene Composites.

[2] Ajayan P.M. (1999). Nanotubes from Carbon. Chem. Rev..

[3] Velasco-Santos C., Martinez-Hernandez A.L., Castano V.M. (2005). Carbon nanotube-polymer nanocomposites: The role of interfaces. Comp. Interface.

[4] Moniruzzaman M., Winey K.I. (2006). Polymer nanocomposites containing carbon nanotubes. Macromolecules.

[5] Ramanathan H., Liu H., Brinson L.C. (2005). Functionalized SWNT/polymer nanocomposites for dramatic property improvement. J. Polym. Sci. B-Polym. Phys..

[6] Meo M., Rossi M. (2006). Prediction of Young's modulus of single wall carbon nanotubes by molecular-mechanics based finite element modelling. Comp. Sci. Technol..

[7] Ritter U., Scharff P., Dmytrenko O.P., Kulish N.P., Prylutskyy Y.I., Grabovskiy Y.E., Belyi N.M., Pinchuk T.N., Alekseev A.N., Sementsov Y.I., Gavrylyuk N.A., Shlapatskaya V.V. (2010). Strength improvement of iPP/MWCNT nanocomposites. Polym. Comp..

[8] Dondero W.E., Gorga R.E. (2006). Morphological and mechanical properties of carbon nanotube/polymer composites via melt compounding. J. Polym. Sci. B-Polym. Phys..

[9] Thomassin J.-M., Huynen I., Jerome R., Detrembleur C. (2010). Functionalized polypropylenes as efficient dispersing agents for carbon nanotubes in a polypropylene matrix; application to electromagnetic interference (EMI) absorber materials. Polymer.

[10] Zhou Z., Wang S.F., Zhang Y., Zhang Y.X. (2006). Effect of different carbon fillers on the properties of PP composites: Comparison of carbon black with multiwalled carbon nanotubes. J. Appl. Polym. Sci..

[11] Baddour C.E., Briens C. (2005). Carbon nanotube synthesis: A review. Int. J. Chem. Reactor Eng..

[12] Koval’chuk A.A., Shevchenko V.G., Shchegolikhin A.N., Nedorezova P.M., Klyamkina A.N., Aladyshev A.M. (2008). Isotactic and syndiotactic polypropylene/multi-wall carbon nanotube composites: Synthesis and properties. J. Mater. Sci..

[13] Logakis E., Pollatos E., Pandis Ch., Peoglos V., Zuburtikudis I., Delides C.G., Vatalis A., Gjoka M., Syskakis E., Viras K., Pissis P. (2010). Structure–property relationships in isotactic polypropylene/multi-walled carbon nanotubes nanocomposites. Comp. Sci. Technol..

[14] Yang B.-X., Shi J.-H., Pramoda K.P., Goh S.H. (2008). Enhancement of the mechanical properties of polypropylene using polypropylene-grafted multiwalled carbon nanotubes. Comp. Sci. Technol..

[15] Masuda J., Torkelson J.M. (2008). Dispersion and major property enhancements in polymer/multiwall carbon nanotube nanocomposites via solid-state shear pulverization followed by melt mixing. Macromolecules.

[16] Xia H., Wang Q., Li K., Hu G.-H. (2004). Preparation of polypropylene/carbon nanotube composite powder with a solid-state mechanochemical pulverization process. J. Appl. Polym. Sci..

[17] Tjong S.C., Liang G.D., Bao S.P. (2007). Electrical behavior of polypropylene/multiwalled carbon nanotube nanocomposites with low percolation threshold. Scripta Mater..

[18] Bikiaris D., Vassiliou A., Chrissafis K., Paraskevopoulos K.M., Jannakoudakis A., Docoslis A. (2008). Effect of acid treated multi-walled carbon nanotubes on the mechanical, permeability, thermal properties and thermo-oxidative stability of isotactic polypropylene. Polym. Degrad. Stab..

[19] Ganβ M., Satapathy B.K., Thunga M., Weidisch R., Pötschke P., Jehnichen D. (2008). Structural interpretations of deformation and fracture behavior of polypropylene/multi-walled carbon nanotube composites. Acta Mater..

[20] Duangphattra N., Aramphongphunb C. (2008). A study of the effects of processing conditions on mechanical properties of polypropylene/multiwall carbon nanotube nanocomposites using design of experiments. Adv. Mater. Res..

[21] Kearns J.C., Shambaugh R.L. (2002). Polypropylene fibers reinforced with carbon nanotubes. J. Appl. Polym. Sci..

[22] Li C., Chou T.W. (2006). Multiscale modeling of compressive behavior of carbon nanotube/polymer composites. Comp. Sci. Technol..

[23] Prashantha K., Soulestin J., Lacrampe M.F., Krawczak P., Dupin G., Claes M. (2009). Masterbatch-based multi-walled carbon nanotube filled polypropylene nanocomposites: Assessment of rheological and mechanical properties. Comp. Sci. Technol..

[24] Zhao P., Wang K., Yang H., Zhang Q., Du R., Fu Q. (2007). Excellent tensile ductility in highly oriented injection-molded bars of polypropylene/carbon nanotubes composites. Polymer.

[25] Mičušík M., Omastová M., Krupa I., Prokeš J., Pissis P., Logakis E., Pandis C., Pötschke P., Pionteck J. (2009). A comparative study on the Electrical and mechanical behaviour of multi-walled carbon nanotube composites prepared by diluting a masterbatch with various types of polypropylenes. J. Appl. Polym. Sci..

[26] Hemmati M., Rahimi G.H., Kaganj A.B., Sepehri S., Rashidi. A.M. (2008). Rheological and mechanical characterization of multi-walled carbon nanotubes/polypropylene nanocomposites. J. Macromol. Sci. B Phys..

[27] Chang T.E., Jensen L.R., Kisliuk A., Pipes R.B., Pyrz R., Sokolov A.P. (2005). Microscopic mechanism of reinforcement in single-wall carbon nanotube/polypropylene nanocomposites. Polymer.

[28] Seidel G.D., Lagouda D.C. (2006). Micromechanical analysis of the effective elastic properties of carbon nanotube reinforced composites. Mech. Mater..

[29] Hammel E., Tang X., Trampert M., Schmitt T., Mauthner K., Eder A., Pötschke P. (2004). Carbon nanofibers for composite applications. Carbon.

[30] Seo M.-K., Lee J.-R., Park S.-J. (2005). Crystallization kinetics and interfacial behaviors of polypropylene composites reinforced with multi-walled carbon nanotubes. Mater. Sci. Eng. A.

[31] Wagner H.D., Lourie O., Feldman Y., Tenne R. (1998). Stress-induced fragmentation of multiwall carbon nanotubes in a polymer matrix. Appl. Phys. Lett..

[32] Satapathy B.K., Ganß M., Weidisch R., Pötschke P., Jehnichen D., Keller T., Jandt K.D. (2007). Ductile-to-semiductile transition in PP-MWNT nanocomposites. Macromol. Rapid Commun..

[33] Yang J., Zhang Z., Friedrich K., Schlarb A.K. (2007). Creep resistant polymer nanocomposites reinforced with multiwalled carbon nanotubes. Macromol. Rapid Commun..

[34] Hine P., Broome V., Ward I. (2005). The incorporation of carbon nanofibres to enhance the properties of self reinforced, single polymer composites. Polymer.

[35] Manchado M.A.L., Valentini L., Biagiotti J., Kenny J.M. (2005). Thermal and mechanical properties of single-walled carbon nanotubes-polypropylene composites prepared by melt processing. Carbon.

[36] Katerelos D.T.G., Joffe R., Labou D., Wallstrom L. (2009). Alteration of the mechanical behaviour of polypropylene owing to successive introduction of multiwall carbon nanotubes and stretching. Mech. Comp. Mater..

[37] Teng C.-C., Ma C.-C.M., Huang Y.-W., Yuen S.-M., Weng C.-C., Chen C.-H., Su S.-F. (2008). Effect of MWCNT content on rheological and dynamic mechanical properties of multiwalled carbon nanotube/polypropylene composites. Comp. A Appl. Sci. Manuf..

[38] Lee G.-W., Jagannathan S., Chae H.G., Minus M.L., Kumar S. (2008). Carbon nanotube dispersion and exfoliation in polypropylene and structure and properties of the resulting composites. Polymer.

[39] Lee S.H., Kim M.W., Kim S.H., Youn J.R. (2008). Rheological and electrical properties of polypropylene/MWCNT composites prepared with MWCNT masterbatch chips. Eur. Polym. J..

[40] Rahmatpour A., Aalaie J. (2008). Steady shear rheological behavior, mechanical properties, and morphology of the polypropylene/carbon nanotube nanocomposites. J. Macromol. Sci. B-Phys..

[41] Pötschke P., Krause B., Stange J., Münstedt H. (2007). Elongational viscosity and foaming behavior of PP modified by electron irradiation or nanotube addition. Macromol. Symp..

[42] Xu D., Wang Z. (2008). Role of multi-wall carbon nanotube network in composites to crystallization of isotactic polypropylene matrix. Polymer.

[43] Grady B.P., Pompeo F., Shambaugh R.L., Resasco D.E. (2002). Nucleation of polypropylene crystallization by single-walled carbon nanotubes. J. Phys. Chem. B.

[44] Bhattacharyya A.R., Sreekumar T.V., Liu T., Kumar S., Ericson L.M., Hauge R.H., Smalley R.E. (2003). Crystallization and orientation studies in polypropylene/single wall carbon nanotube composite. Polymer.

[45] Assouline E., Lustiger A., Barber A.H., Cooper C.A., Klein E., Wachtel E., Wagner H.T. (2003). Nucleation ability of multiwall carbon nanotubes in polypropylene composites. J. Polym. Sci. B-Polym. Phys..

[46] Valentini L., Biagiotti J., Kenny J.M., Santucci S. (2003). Morphological characterization of single-walled carbon nanotubes-PP composites. Comp. Sci. Technol..

[47] Leelapornpisit W., Ton-That M., Perrin-Sarazin F., Cole K.C., Denault J., Simard B. (2005). Effect of carbon nanotubes on the crystallization and properties of polypropylene. J. Polym. Sci. B- Polym. Phys..

[48] Wang K., Tang C., Zhao P., Yang H., Zhang Q., Du R., Fu Q. (2007). Rheological investigations in understanding shear-enhanced crystallization of isotactic poly(propylene)/multi-walled carbon nanotube composites. Macromol. Rapid Commun..

[49] Kaganj A.B., Rashidi A.M., Arasteh R., Taghipoor S. (2009). Crystallisation behaviour and morphological characteristics of poly(propylene)/multi-walled carbon nanotube nanocomposites. J. Experim. Nanosci..

[50] Reyes-de Vaaben S., Aguilar A., Avalos F., Ramos-de Valle L.F. (2008). Carbon nanoparticles as effective nucleating agents for polypropylene. J. Therm. Anal. Cal..

[51] Avila-Orta C.A., Medellín-Rodríguez F.J., Dávila-Rodríguez M.V., Aguirre-Figueroa Y.A., Yoon K., Hsiao B.S. (2007). Morphological features and melting behavior of nanocomposites based on isotactic polypropylene and multiwalled carbon nanotubes. J. Appl. Polym. Sci..

[52] Peneva Y., Valcheva M., Minkova L., Miušík M., Omastová M. (2008). Nonisothermal crystallization kinetics and microhardness of PP/CNT composites. J. Macromol. Sci. B-Phys..

[53] Hou Z., Wang K., Zhao P., Zhang Q., Yang C., Chen D., Du R., Fu Q. (2008). Structural orientation and tensile behavior in the extrusion-stretched sheets of polypropylene/multi-walled carbon nanotubes' composite. Polymer.

[54] Jose M.V., Dean D., Tyner J., Price G., Nyairo E. (2007). Polypropylene/carbon nanotube nanocomposite fibers: Process-morphology- property relationships. J. Appl. Polym. Sci..

[55] Anand K.A., Agarwal U.S., Joseph R. (2006). Carbon nanotubes induced crystallization of poly(ethylene terephthalate). Polymer.

[56] Causin V., Yang B.X., Marega C., Goh S.H., Marigo A. (2009). Nucleation, structure and lamellar morphology of isotactic polypropylene filled with polypropylene-grafted multiwalled carbon nanotubes. Eur. Polym. J..

[57] Fereidoon A., Ahangari M.G., Saedodin S. (2009). A DSC study on the nonisothermal crystallization kinetics of polypropylene/single-walled carbon nanotube nanocomposite. Polym. Plast. Techn. Eng..

[58] Lu K., Grossiord N., Koning C.E., Miltner H.E., van Mele B., Loos J. (2008). Carbon nanotube/isotactic polypropylene composites prepared by latex technology: Morphology analysis of CNT-induced nucleation. Macromolecules.

[59] Zhang S., Minus M.L., Zhu L., Wong C.-P., Kumar S. (2008). Polymer transcrystallinity induced by carbon nanotubes. Polymer.

[60] Valentini L., Biagiotti J., Kenny J.M., Santucci S. (2003). Effects of single-walled carbon nanotubes on the crystallization behavior of polypropylene. J. Appl. Polym. Sci..

[61] Miltner H.E., Grossiord N., Lu K., Loos J., Koning C.E., Van Mele B. (2008). Isotactic polypropylene/carbon nanotube composites prepared by latex technology. Thermal analysis of camon nanotube induced nucleation. Macromolecules.

[62] Yin C.L., Liu Z.Y., Yang W., Yang M.B., Feng J.M. (2009). Crystallization and morphology of iPP/MWCNT prepared by compounding iPP melt with MWCNT aqueous suspension. Coll. Polym. Sci..

[63] Wu D.F., Sun Y.R., Wu L., Zhang M. (2008). Linear viscoelastic properties and crystallization behavior of multi-walled carbon nanotube/ polypropylene composites. J. Appl. Polym. Sci..

[64] Deng H., Zhang R., Bilotti E., Loos J., Peijs T. (2009). Conductive polymer tape containing highly oriented carbon nanofillers. J. Appl. Polym. Sci..

[65] Kharchenko S.B., Douglas J.F., Obrzut J., Grulke E.A., Migler K.B. (2004). Flow-induced properties of nanotube-filled polymer materials. Nat. Mater..

[66] Li C., Liang T., Lu W., Tang C., Hu X., Cao M., Liang J. (2004). Improving the antistatic ability of polypropylene fibers by inner antistatic agent filled with carbon nanotubes. Comp. Sci. Technol..

[67] Andrews R., Jacques D., Minot M., Rantell T. (2002). Fabrication of carbon multiwall nanotube/polymer composites by shear mixing. Macromol. Mater. Eng..

[68] Deng H., Skipa T., Zhang R., Lellinger D., Bilotti E., Alig I., Peijs T. (2009). Effect of melting and crystallization on the conductive network in conductive polymer composites. Polymer.

[69] Seo M.K., Park S.J. (2004). Electrical resistivity and rheological behaviors of carbon nanotubes-filled polypropylene composites. Chem. Phys. Lett..

[70] Du F.M., Scogna R.C., Zhou W., Brand S., Winey K.I. (2004). Nanotube networks in polymer nanocomposites: Rheology and electrical conductivity. Macromolecules.

[71] Alig I., Lellinger D., Dudkin S.M., Pötschke P. (2007). Conductivity spectroscopy on melt processed polypropylene-multiwalled carbon nanotube composites: Recovery after shear and crystallization. Polymer.

[72] Immonen K., Nättinen K., Sarlin J., Hartikainen J. (2009). Conductive plastics with hybrid materials. J. Appl. Polym. Sci..

[73] Liao S.-H., Yen C.-Y., Weng C.-C., YLin F., Ma C.-C.M., Yang C.-H., Tsai M.-C., Yen M.-Y., Hsiao M.-C., Lee S.-J., Xie X.-F., Hsiao Y.-H. (2008). Preparation and properties of carbon nanotube/polypropylene nanocomposite bipolar plates for polymer electrolyte membrane fuel cells. J. Power Sources.

[74] Liang G.D., Bao S.P., Tjong S.C. (2007). Mechanical behaviors of polypropylene/carbon nanotube nanocomposites: The effects of loading rate and temperature. Mater. Sci. Eng. B.

[75] Bao H.-D., Guo Z.-X., Yu J. (2008). Effect of electrically inert particulate filler on electrical resistivity of polymer/multi-walled carbon nanotube composites. Polymer.

[76] Lozano K., Barrera E.V. (2001). Nanofiber-reinforced thermoplastic composites. I. Thermoanalytical and mechanical analyses. J. Appl. Polym. Sci..

[77] Yang J., Lin Y., Wang J., Lai M., Li J., Liu J., Tong X., Cheng H. (2005). Morphology, thermal stability, and dynamic mechanical properties of atactic polypropylene/carbon nanotube composites. J. Appl. Polym. Sci..

[78] Seo M.K., Park S.J. (2004). A kinetic study on the thermal degradation of multi-walled carbon nanotubes-reinforced poly(propylene) composites. Macromol. Mater. Eng..

[79] Marosföi B.B., Szabó A., Marosi G., Tabuani D., Camino G., Pagliari S. (2006). Thermal and spectroscopic characterization of polypropylene-carbon nanotube composites. J. Therm. Anal. Cal..

[80] Vladimirov V., Betchev C., Vassiliou A., Papageorgiou G., Bikiaris D. (2006). Dynamic mechanical and morphological studies of isotactic polypropylene/fumed silica nanocomposites with enhanced gas barrier properties. Comp. Sci. Technol..

[81] Vassiliou A., Bikiaris D., Pavlidou E. (2007). Optimizing melt-processing conditions for the preparation of iPP/fumed silica nanocomposites: Morphology, mechanical and gas permeability properties. Macromol. React. Eng..

[82] Kashiwagi T., Grulke E., Hilding J., Harris R.H., Awad W.H., Dougls J. (2002). Thermal degradation and flammability properties of poly(propylene)/carbon nanotube composites. Macromol. Rapid Commun..

[83] Kashiwagi T., Grulke E., Hilding J., Groth K., Harris R., Butler K., Shields J., Kharchenko S., Douglas J. (2004). Thermal and flammability properties of polypropylene/carbon nanotube nanocomposites. Polymer.

[84] Bom D., Andrews R., Jacques D., Anthony J., Chen B., Meier M.S., Selegue J.P. (2002). Thermogravimetric Analysis of the Oxidation of Multiwalled Carbon Nanotubes: Evidence for the Role of Defect Sites in Carbon Nanotube Chemistry. Nano Lett..

[85] Andrews R., Jacques D., Qian D., Dickey E.C. (2001). Purification and structural annealing of multiwalled carbon nanotubes at graphitization temperatures. Carbon.

[86] Moore E.M., Ortiz D.L., Marle V.T., Shambaugh R.L., Grady B.P. (2004). Enhancing the strength of polypropylene fibers with carbon nanotubes. J. Appl. Polym. Sci..

[87] Xiao Y., Zhang X., Cao W., Wang K., Tan H., Zhang Q., Du R., Fu Q. (2007). Dispersion and mechanical properties of polypropylene/multiwall carbon nanotubes composites obtained via dynamic packing injection molding. J. Appl. Polym. Sci..

[88] Liao S.H., Weng C.C., Yen C.Y., Hsiao M.C., Ma C.C.M., Tsai M.C., Su A., Yen M.Y., Lin Y.F., Liu P.L. (2010). Preparation and properties of functionalized multiwalled carbon nanotubes/polypropylene nanocomposite bipolar plates for polymer electrolyte membrane fuel cells. J. Power Sources.

[89] McIntosh D., Khabashesku V.N., Barrera E.V. (2006). Nanocomposite fiber systems processed from fluorinated single-walled carbon nanotubes and a polypropylene matrix. Chem. Mater..

[90] Zhou Z., Wang S., Lu L., Zhang Y., Zhang Y. (2007). Isothermal crystallization kinetics of polypropylene with silane functionalized multi-walled carbon nanotubes. J. Polym. Sci. B-Polym. Phys..

[91] Kelarakis A., Yoon K., Sics I., Somani R.H., Chen X.M., Hsiao B.S., Chu B. (2006). Shear-induced orientation and structure development in isotactic polypropylene melt containing modified carbon nanofibers. J. Polym. Sci. B-Polym. Phys..

[92] Ristolainen N., Vainio U., Paavola S., Torkkeli M., Serimaa R., Seppälä J. (2005). Polypropylene/organoclay nanocomposites compatibilized with hydroxyl-functional polypropylenes. J. Polym. Sci. B- Polym. Phys..

[93] Xu D.-H., Wang Z.-G. (2008). Influence of carbon nanotube aspect ratio on normal stress differences in isotactic polypropylene nanocomposite melts. Macromolecules.

[94] Chen X.H., Hu J., Li W.H., Liu Y.Q., Chen C.S., Wang Y.G. (2008). Preparation and crystallization of carbon nanotube/maleic anhydride-grafted polypropylene composites. J. Mater. Sci. Technol..

[95] Li W.-H., Chen X.-H., Yang Z., Xu L.-S. (2009). Structure and properties of polypropylene-wrapped carbon nanotubes composite. J. Appl. Polym. Sci..

[96] Zhou Z., Wang S., Lu L., Zhang Y., Zhang Y. (2008). Functionalization of multi-wall carbon nanotubes with silane and its reinforcement on polypropylene composites. Comp. Sci. Technol..

[97] Koval’chuk A.A., Shevchenko V.G., Shchegolikhin A.N., Nedorezova P.M., Klyamkina A.N., Aladyshev A.M. (2008). Effect of carbon nanotube functionalization on the structural and mechanical properties of polypropylene/MWCNT composites. Macromolecules.

[98] Vaisman L., Marom G., Wagner H.D. (2006). The role of surfactants in dispersion of carbon nanotubes. Adv. Funct. Mater..

[99] Garg A., Sinnott S.B. (1998). Effect of chemical functionalization on the mechanical properties of carbon nanotubes. Chem. Phys. Lett..

[100] Lee S. H., Cho E. N., Jeon S. H., Youn J. R. (2007). Rheological and electrical properties of polypropylene composites containing functionalized multi-walled carbon nanotubes and compatibilizers. Carbon.

[101] Ahangari M.G., Fereidoon A., Saedodin S. (2008). Mechanical and thermal properties of PP/compatibilized PP/acid treated SWCNTs nanocomposites: Effect of different acid treatment times. E-Polymers.

[102] Wu D., Sun Y., Zhang M. (2009). Kinetics study on melt compounding of carbon nanotube/polypropylene nanocomposites. J. Polym. Sci. B-Polym. Phys..

[103] Jin S.H., Kang C.H., Yoon K.H., Bang D.S., Park Y.-B. (2009). Effect of compatibilizer on morphology, thermal, and rheological properties of polypropylene/functionalized multi-walled carbon nanotubes composite. J. Appl. Polym. Sci..

[104] Prashantha K., Soulestin J., Lacrampe M.F., Claes M., Dupin G., Krawczak P. (2008). Multi-walled carbon nanotube filled polypropylene nanocomposites based on masterbatch route: Improvement of dispersion and mechanical properties through PP-g-MA addition. eXPRESS Polym. Lett..

[105] Velasco-Santos C., Martinez-Hernandez A.L., Fisher F.T., Ruoff R., Castano V.M. (2003). Improvement of Thermal and Mechanical Properties of Carbon Nanotube Composites through Chemical Functionalization. Chem. Mater..

[106] Philip B., Xie J., Abraham J.K., Varadan V.K. (2005). Polyaniline/carbon nanotube composites: Starting with phenylamino functionalized carbon nanotubes. Polym. Bull..

[107] Wiemann K., Kaminsky W., Gojny F.H., Schulte K. (2005). Synthesis and properties of syndiotactic poly(propylene)/carbon nanofiber and nanotube composites prepared by *in situ* polymerization with metallocene/MAO catalysts. Macromol. Chem. Phys..

[108] Kaminsky W., Wiemann K. (2006). Polypropene nanocomposites by metallocene/MAO catalysts. Comp. Interf..

[109] Kaminsky W., Funck A., Wiemann K. (2006). Nanocomposites by *in situ* polymerization of olefins with metallocene catalysts. Macromol. Symp..

[110] Funck A., Kaminsky W. (2007). Polypropylene carbon nanotube composites by *in situ* polymerization. Comp. Sci. Technol..

[111] Bonduel D., Bredeau S., Alexandre M., Monteverde F., Dubois P. (2007). Supported metallocene catalysis as an efficient tool for the preparation of polyethylene/carbon nanotube nanocomposites: Effect of the catalytic system on the coating morphology. J. Mater. Chem..

[112] Bredeau S., Boggioni L., Bertini F., Tritto I., Monteverde F., Alexandre M., Dubois P. (2007). Ethylene-norbornene copolymerization by carbon nanotube-supported metallocene catalysis: Generation of high-performance polyolefinic nanocomposites. Macromol. Rapid Commun..

[113] Kaminsky W., Funck A. (2007). *In situ* polymerization of olefins with nanoparticles by metallocene-catalysis. Macromol. Symp..

[114] Koval’chuk A.A., Shchegolikhin A.N., Shevchenko V.G., Nedorezova P.M., Klyamkina A.N., Aladyshev A.M. (2008). Synthesis and properties of polypropylene/multiwall carbon nanotube composites. Macromolecules.

[115] Auriemma F., De Rosa C. (2003). New Concepts in Thermoplastic Elastomers: The Case of Syndiotactic Polypropylene, an Unconventional Elastomer with High Crystallinity and Large Modulus. J. Am. Chem. Soc..

[116] Gorrasi G., Romeo V., Sannino D., Sarno M., Ciambelli P., Vittoria V., De Vivo B., Tucci V. (2007). Carbon nanotube induced structural and physical property transitions of syndiotactic polypropylene. Nanotechnology.

[117] Sarno M., Gorrasi G., Sannino D., Sorrentino A., Ciambelli P., Vittoria V. (2004). Polymorphism and thermal behaviour of syndiotactic poly(propylene)/carbon nanotube composites. Macromol. Rapid Commun..

[118] Wu M., Shaw L. (2006). Electrical and mechanical behaviors of carbon nanotube-filled polymer blends. J. Appl. Polym. Sci..

[119] Zhang L., Wan C., Zhang Y. (2009). Morphology and electrical properties of polyamide 6/polypropylene/multi-walled carbon nanotubes composites. Comp. Sci. Technol..

[120] Zou J., Zhang Y., Juang J., Wu H., Qiu Y.P. (2009). Preparation and properties of PP/PLA/multiwall carbon nanotube composites filaments obtained by melt compounding. Mater. Sci. Forum.

[121] Valentini L., Biagiotti J., Kenny J.M., Manchado M.A.L. (2003). Physical and mechanical behavior of single-walled carbon nanotube/polypropylene/ethylene-propylene-diene rubber nanocomposites. J. Appl. Polym. Sci..

[122] Liu L., Wang Y., Li Y., Wu J., Zhou Z., Jiang C. (2009). Improved fracture toughness of immiscible polypropylene/ethylene-co-vinyl acetate blends with multiwalled carbon nanotubes. Polymer.

[123] Khare R.A., Bhattacharyya A.R., Kulkarni A.R., Saroop M., Biswas A. (2008). Influence of multiwall carbon nanotubes on morphology and electrical conductivity of PP/ABS blends. J. Polym. Sci. B-Polym. Phys..

